# *pix-1* Controls Early Elongation in Parallel with *mel-11* and *let-502* in *Caenorhabditis elegans*

**DOI:** 10.1371/journal.pone.0094684

**Published:** 2014-04-14

**Authors:** Emmanuel Martin, Sharon Harel, Bernard Nkengfac, Karim Hamiche, Mathieu Neault, Sarah Jenna

**Affiliations:** Department of Chemistry, Pharmaqam, Biomed, Université du Québec à Montréal (UQÀM), Montréal, Québec, Canada; University of North Carolina at Chapel Hill, United States of America

## Abstract

Cell shape changes are crucial for metazoan development. During *Caenorhabditis elegans* embryogenesis, epidermal cell shape changes transform ovoid embryos into vermiform larvae. This process is divided into two phases: early and late elongation. Early elongation involves the contraction of filamentous actin bundles by phosphorylated non-muscle myosin in a subset of epidermal (hypodermal) cells. The genes controlling early elongation are associated with two parallel pathways. The first one involves the *rho-1/*RHOA-specific effector *let-502/*Rho-kinase and *mel-11*/myosin phosphatase regulatory subunit. The second pathway involves the CDC42/RAC-specific effector *pak-1*. Late elongation is driven by mechanotransduction in ventral and dorsal hypodermal cells in response to body-wall muscle contractions, and involves the CDC42/RAC-specific Guanine-nucleotide Exchange Factor (GEF) *pix-1*, the GTPase *ced-10/*RAC and *pak-1*.

In this study, *pix-1* is shown to control early elongation in parallel with *let-502/mel-11*, as previously shown for *pak-1*. We show that *pix-1*, *pak-1* and *let-502* control the rate of elongation, and the antero-posterior morphology of the embryos. In particular, *pix-1* and *pak-1* are shown to control head, but not tail width, while *let-502* controls both head and tail width. This suggests that *let-502* function is required throughout the antero-posterior axis of the embryo during early elongation, while *pix-1/pak-1* function may be mostly required in the anterior part of the embryo. Supporting this hypothesis we show that low *pix-1* expression level in the dorsal-posterior hypodermal cells is required to ensure high elongation rate during early elongation.

## Introduction

In mammals, the CDC42/RAC-specific Guanine-nucleotide exchange factor (GEF) α/β-PIX and the CDC42/RAC-specific effector kinase PAKs were shown to control cell migration, cell polarity, cytoskeleton remodeling and focal adhesion complex assembly/disassembly dynamics [1]. Their involvement in the control of epithelium morphogenesis and migration of epithelial sheets has also been recently established in mammals [2], and in model organisms such as *Drosophila melanogaster* and *Caenorhabditis elegans* [3]–[5]. Study of epithelial morphogenesis in *C. elegans* appears as an excellent model to better understand the function of α/β-PIX and PAKs during complex morphogenic events in living organisms.

In the nematode *C. elegans*, embryonic elongation involves the extension of the embryo along its longitudinal axis and a reduction of its transverse diameter, resulting in a 4-fold increase in length. This morphogenetic event involves dramatic changes in the shape of the epidermal (hypodermal) cells. Elongation is divided into an early and a late phase. The early phase, from comma to 1.75-fold stage – corresponding to embryos that are 1.75-fold in length compared to non-elongated embryos –, occurs through contraction of filamentous actin bundles (FBs) in hypodermal cells [6]. The hypodermis is composed of ventral, lateral (seam cells) and dorsal cells, which are linked by adherens junctions [7]. Contraction of FBs during early elongation is thought to be high in the seam cells and low in dorsal and ventral hypodermal cells [6], [8].

The late phase of elongation involves mechanotransduction signaling from the body-wall muscles to the dorsal and ventral hypodermal cells [4]. At the 1.5-fold stage of development, muscle cells form connections, called trans-epidermal attachment structures (TEAs), with the dorsal and ventral hypodermis [9]. As embryos develop to the 1.75-fold stage, the muscles become functional and start contracting, thus inducing chemical changes in the overlying hypodermal cells through mechanical tension applied on the TEAs [4].

The signal transduction pathways that regulate early and late elongation have been extensively investigated over the last 15 years. Interestingly, many genes controlling morphological changes of the hypodermis during elongation are effectors or regulators of Rho GTPases [3], [4], [10], [11]. Rho GTPases are molecular switches controlling a wide-range of cellular functions involving cell shape changes, cell migration, cell proliferation and differentiation [12]. They cycle between an “ON” GTP-bound form and an “OFF” GDP-bound form. When bound to GTP, they interact with specific effectors. They are regulated by three families of proteins: Guanine nucleotide-Exchange Factors (GEFs); GTPase-Activating Proteins (GAPs); and Guanine nucleotide-Dissociation Inhibitors (GDIs). To date, although three Rho GTPases (*rho-1/*RHOA, *ced-10/*RAC and *mig-2*/RHOG) have been implicated in pathways controlling elongation, only three of their regulators (GAPs and GEFs) have been shown to be involved in this process [4], [10], [11], [13], suggesting that others remain to be identified.

In hypodermal cells, contraction of the FBs during early elongation depends on the regulation of myosin-light-chain (MLC-4/MLC) phosphorylation by three serine-threonine kinases, the RHO-1/RHOA-effector kinase LET-502/ROCK, the *C. elegans* ortholog of the CDC42-effector myotonic dystrophy kinase MRCK-1/MRCK and the CDC42/RAC-effector kinase PAK-1/PAK1. These kinases act antagonistically with the MEL-11/PP-1M and are organized in two parallel pathways: The *let-502/mel-11* pathway including *mrck-1* and a second pathway involving *pak-1* [3], [8], [10]. Downstream of these pathways, MLC-4/MLC phosphorylation leads to non-muscle myosin filament assembly and contractility, while its dephosphorylation is associated with relaxation.

LET-502/ROCK is an essential component of the *let-502/mel-11* pathway and an essential regulator of elongation [14]. It is activated downstream of the Rho GTPase RHO-1/RHOA [15], that may itself be activated by the GEF RHGF-2 [13] and inactivated by the GAP RGA-2 [11]. Inactivation of RHO-1 by RGA-2, occurs in ventral and dorsal hypodermal cells during early elongation leading to inactivation of LET-502/ROCK and reduction of FB contractions in these cells [11]. RHO-1/RHOA and LET-502/ROCK may then be activated in the lateral hypodermal cells where most of the FB contractions may occur during early elongation. Consistent with this model, expression of MLC-4/MLC in the lateral cells can rescue *mlc-4 loss-of-function*-associated elongation defects, while expression of MLC-4/MLC in ventral and dorsal cells cannot [3]. In seams cells, MEL-11/PP-1M may be inhibited by LET-502/ROCK and MRCK-1/MRCK presumably through phosphorylation [3], [8].

The second pathway involves the CDC42/RAC-effector PAK-1, the PP2C phosphatase FEM-2/POPX2 and a RHO/RAC-specific GTP-nucleotide exchange factor (GEF) UNC-73/TRIO. The function of these two later proteins in the regulation of MLC-4 phosphorylation and/or PAK-1 function remains unknown [3], [8], [10], [16].

To date, the genes controlling the *pak-1* pathway during early elongation remain unknown. Moreover, the biological significance of the functional redundancy of the *mel-11/let-502* and *pak-1* pathways is not clear. This redundancy is intriguing since it does not appear to add robustness to the elongation system: a single perturbation in any component of the *mel-11/let-502* pathway induces a high proportion of embryonic lethality [10]. This suggests that the *mel-11/let-502* and *pak-1* pathways have unique functions during elongation that remain to be identified.

The CDC42/RAC-specific GEF, PIX is a well-known activator of PAKs in several organisms [1]. In *C. elegans*, *pix-1* codes for a protein homologous to the mammalian β-PIX (Figure S1). It contains a Src-homology 3 (SH3); a GEF/dbl homology (DH); a GIT-binding (GBD), and a PDZ binding (ZB) domains (Figure S1). These conserved domains are shown in mammals to mediate β-PIX interaction with PAK1-3, the Rho GTPases CDC42 and RAC, the phosphatases POPX1/2, the ARFGAP GIT, the tumor suppressor Scribble and the postsynaptic density protein Shank [1]. In *C. elegans*, PIX-1 was shown to activate PAK-1 in a GTPase-independent manner in migrating distal tip cells (DTC) during gonad morphogenesis in larvae [17]. In this system, PAK-1 activation still appears to be at least partially dependent on CED-10/RAC [18]. PIX-1 was also shown to activate PAK-1 through the GTPase CED-10/RAC in hypodermal cells during late elongation of embryos [4].

In this study, we demonstrate that *pix-1* controls early elongation in parallel with *mel-11/let-502*. Our data suggest that *pix-1* is a novel component of the *pak-1* pathway while retaining some function during early elongation independent from *pak-1*. We show that the *pix-1*/*pak-1* pathway controls the antero-posterior morphology of the embryo during early elongation through regulating head width, while *let-502*/ROCK controls both the head and tail width. Our study proposes a novel model for early elongation where the *mel-11/let-502* and *pix-1*/*pak-1* pathways have redundant and complementary functions to shape the antero-posterior axis of the embryo.

## Results

### *pix-1* and *pak-1* control early elongation

**Figure 1:**
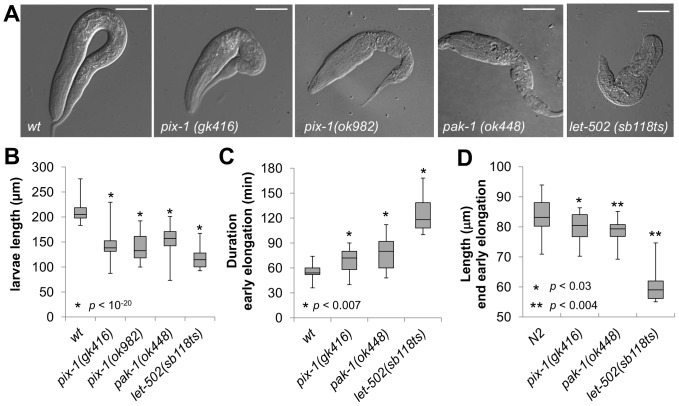
*pix-1* and *pak-1* control early elongation. A) Arrested larvae of *pix-1(gk41*6), *pix-1(ok982)*, *pak-1(ok448)* and *let-502(sb118ts)* mutants grown at 25.5°C. Bar = 25 µm. B) Box-plot representing the distribution of sizes of arrested larvae in mutant populations grown at 25.5°C. The box-plot represents the min, max, 25^th^, 50^th^ (median) and 75^th^ percentile of the population. Distribution of *wild-type* animals (*wt*) has been established using N2 L1 larvae synchronized by starvation after hypochlorite treatment. C) Box-plot representing the distribution of the duration in minutes of early elongation for *wt* and mutants embryos. Embryos are collected through dissection of hermaphrodites grown at 25.5°C. Embryonic development is recorded at 23–24°C. D) Box-plot representing the distribution of the length of embryos (in μm) at the end of early elongation. The same population of embryos was used to generate data presented in panel C and D. Student's T-test *p-values* are indicated.

To investigate the role of *pix-1* during early elongation, we examined the phenotypes of *pix-1(gk416)* and *pix-1(ok982)* embryos. As controls, we characterized the elongation phenotypes of *pak-1(ok448)* and *let-502(sb118ts)* mutant embryos, which display early elongation defects [3], [10]. *pix-1(gk416)* and *pak-1(ok448)* are null alleles [3], [17]. *pix-1(ok982)* contains a 1002 bp deletion spanning exons 8 to 11 and may code for a protein that retains the N-terminal SH3 and RhoGEF/DH domains of PIX-1 (Figure S1). *let-502(sb118ts*) is a thermosensitive allele coding for the RHO-1/RHOA-effector kinase LET-502/ROCK. This allele shows no obvious phenotypes at 20°C, but displays strong elongation defects and L1-larval arrest characteristic of strong hypomorphic and null *let-502* alleles at 25.5°C [10] (Figure 1).

Phenotypic characterisation of animals carrying *pix-1(gk416), pix-1(ok982)* and *pak-1(ok448)* alleles revealed low penetrance embryonic lethality (Emb) and early larval arrest (Lva) confirming previous findings (Table 1) [4]. Measurements of *pix-1(gk416), pix-1(ok982), pak-1(ok448)* and *let-502(sb118ts)* arrested larvae showed that they were significantly shorter than synchronized *wt* L1 larvae at 25.5°C (T-test, *p<0.01*) (Figure 1A and B). To better characterize the elongation defects associated with the *pix-1*, *pak-1* and *let-502* alleles, we measured the duration of early elongation using four-dimensional light microscopy. To do so, embryos were collected through dissection of hermaphrodites grown at 25.5°C, and embryonic development was imaged at 23–24°C. The duration of early elongation was measured from the 1.2-fold stage to the beginning of late elongation – when body wall muscles started contracting (Figure 1C). Animals carrying *let-502(sb118ts)* (n = 10), *pix-1(gk416)* (n = 25) and *pak-1(ok448)* (n = 20) alleles developed significantly slower than *wt* animals (n = 17) during early elongation (Figure 1C). We also measured the length of the embryos at the end of early elongation (Figure 1D), and found that mutant embryos were significantly shorter than *wt* embryos (Figure 1D). These data demonstrate that the elongation rate of *pix-1*, *pak-1* and *let-502* mutant embryos is significantly reduced during early elongation, and provide the first evidence of a requirement for *pix-1* at this stage. They also confirm the involvement of *pak-1* during early elongation [3].

**Table 1:** Embryonic lethality and arrest of non-elongated larvae in *pix-1 and pak-1* mutants.

	18°C			25.5°C		
allele	Emb (%)	Lva (%)	n	Emb (%)	Lva (%)	n
*wt*	0	0	709	0.5	0	2367
*pix-1 (ok982)*	6	7	1140	11	15	679
*pix-1 (gk416)*	1	6	713	2.14	8.5	1216
*pix-1(gk416);sajEx1[pix-1p::pix-1::GFP; rol-6]*	-	-	-	1.2	2.0	917
*pak-1(ok448)*	1	12	2502	3	25.6	264

**Figure 2:**
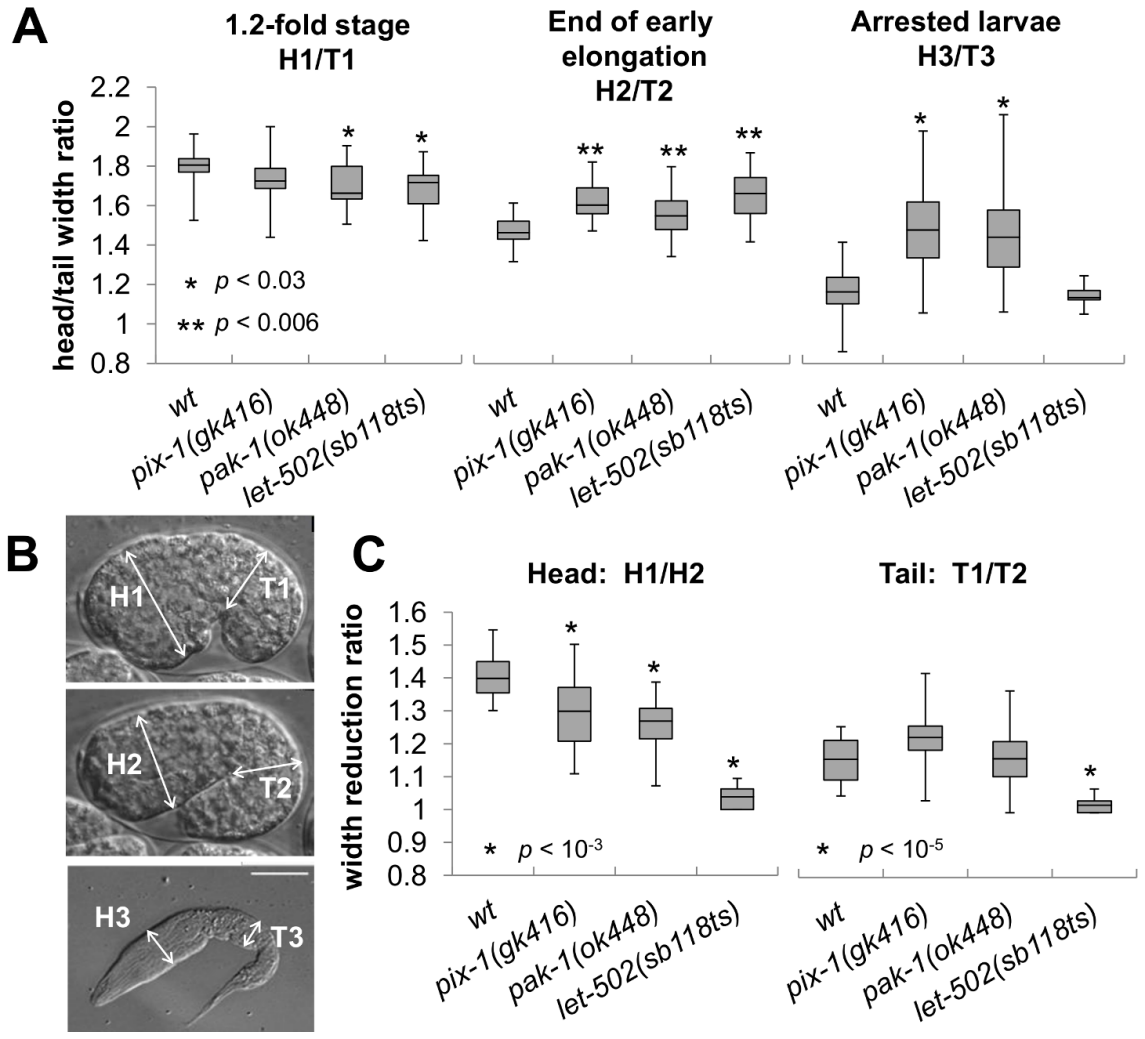
*pix-1, pak-1* and *let-502* control the head to tail width ratio of elongating embryos. Head (H) and tail (T) width are measured on 1.2-fold stage embryos (H1 and T1); at the end of early elongation (H2 and T2) and in arrested larvae (H3 and T3). In all panels of this figure animals were grown at 25.5°C. Embryos were collected through dissection of hermaphrodite grown at 25.5°C. Embryonic development is then recorded using 4-dimensional microscopy at 23–24°C. A) Distribution of ratio between the head and tail width of embryos at 1.2-fold stage (H1/T1; left panel), at the end of early elongation (H2/T2; middle panel) and of arrested larvae (H3/T3; right panel) in *wt*, *pix-1(gk416)*, *pak-1(ok448)* and *let-502(sb118ts)* mutants. B) Localisation of measured areas in embryos and larvae C) Distribution of the head (H1/H2) and tail (T1/T2) width reduction ratios during early elongation. The box-plots represent the min, max, 25^th^, 50^th^ (median) and 75^th^ percentiles of the populations. Student's T-test *p-values* are indicated

Interestingly, *pix-1* and *pak-1* arrested larvae had very similar morphologies and behavior. For example, they were thin and clear when compared to *let-502* arrested larvae (Figure 1A). Furthermore, they appeared to have different antero-posterior morphologies when compared to *wt* and *let-502* larvae. When the width of the head (H3, Figure 2A and B) and the width of the tail (T3, Figure 2A and B) were measured and combined as a head/tail width ratio (H3/T3, Figure 2B), *pix-1(gk416), pix-1(ok982)* and *pak-1(ok448)* had significantly higher ratios than *wt* and *let-502* larvae (H3/T3, Figure 2B, data not shown). This indicates that *pix-1* and *pak-1* mutant arrested larvae present a morphological alteration characterized by an inflated anterior part of their body when compared to their posterior part. These mutants arrested larvae also had severe pharynx pumping defects and were completely paralyzed, although pumping in L3 escapers was similar to *wt* larvae (Figure S2). While these later phenotypes may be physiological consequences of primary developmental phenotypes, they support the fact that a similar array of phenotypes is observed in *pix-1* and *pak-1* mutant animals. These data support then the hypothesis that *pix-1* and *pak-1* could be part of the same developmental pathway. This also suggests that *pix-1* and *pak-1* control the relative width of the head vs. the tail of developing embryos, a process that does not seem to require *let-502*.

### *pix-1* and *pak-1* control the head to tail width of the embryos during early elongation

Since *pix-1* and *pak-1*are also involved in the control of late elongation, we investigated whether their function during early elongation is required to control the head to tail width of the animal. To do so, we measured the H/T ratio of *pix-1(gk416)*, *pak-1(ok448)* and *let-502(sb118ts)* embryos at the beginning, 1.2-fold stage (H1/T1, Figure 2A and B), and at the end of the early elongation (H2/T2, Figure 2A and B). These experiments were done using the same populations of embryos characterized previously (Figure 1C and D). While no change, or a reduced H/T ratio was observed in *pix-1, pak-1* and *let-502* mutants when compared to *wt* animals at the beginning of early elongation (H1/T1, Figure 2A), all three mutants showed a significantly higher H/T ratio than *wt* embryos at the end of early elongation (T-test *p-values*<0.006; H2/T2, Figure 2A). This shows that the inflated head vs tail morphology observed in *pix-1* and *pak-1* arrested larvae is also observed at the end of early elongation in *pix-1*, *pak-1* and *let-502* mutant embryos, suggesting that *pix-1*, *pak-1* and *let-502* control the head to tail width of the embryos at that stage. We then measured the reduction of head (H) and tail (T) width during early elongation. To do so, we compared the width of the head and the width of the tail of the embryos at the beginning and the end of early elongation (H1/H2 and T1/T2 respectively; Figure 2C). In *wt* embryos the head and the tail appeared to be 1.4- and 1.16-fold wider at the beginning than at the end of early elongation (Figure 2C). This shows that the head width reduces more than the tail width during early elongation in *wt* animals. We found that the head width of the *pix-1* and *pak-1* mutant embryos reduced significantly less during early elongation than *wt* embryos (Figure 2C left panel). We also found that the tail width reduced similarly in *pix-1* and *pak-1* mutant embryos than in *wt* embryos. Interestingly, the degrees of head and tail width reduction in *let-502(sb118ts)* embryos were significantly lower than *wt* embryos and not significantly different than 1, suggesting that in *let-502* mutant animals neither the head, nor the tail width of the animal reduce during early elongation (Figure 2C).

Together, these data suggest that the higher reduction of the head versus the tail width of the embryo during early elongation requires *pix-1*, *pak-1* and *let-502* function. These three genes appear to be required for reduction of the head width of the embryo, while only *let-502* may be required for reduction of the tail width.

### *pix-1* and *pak-1* control early elongation in parallel with *mel-11/let-502* pathway

*pak-1* was previously proposed to control early elongation in parallel with the *mel-11/let-502* pathway [3]. To assess whether *pix-1* also controls early elongation in parallel with the *mel-11/let-502* pathway, we tested whether *pix-1(gk416)* genetically interacts with *mel-11(it26)* and *let-502(sb118ts)* temperature sensitive mutants, as reported for *pak-1* [3]. At 18°C, 98% of *mel-11(it26)* mutant embryos rupture during elongation as previously described (Table 2, line 3) [10]. The penetrance of this phenotype is increased to 100% at 25.5°C. As previously shown, *mel-11(it26); pak-1(ok448)* double mutant embryos display phenotypes similar to *mel-11(it26)* at both 18°C and 25.5°C (Table 2 line 10) [3]. Interestingly, the *mel-11(it26)*-associated embryonic rupturing is completely suppressed by *pix-1(gk416)* at 18°C and 25.5°C (Table 2, line 4). A small fraction of *mel-11(it26); pix-1(gk416)* hatched larvae arrest at the L1 stage with characteristic *pix-1* phenotypes, and a penetrance similar to *pix-1(gk416)* at 18°C (Table 2, line 2 and 4). Surprisingly, at 25.5°C, 9.7% of *mel-11(it26); pix-1(gk416)* embryos arrest between 1.2- and 2-fold stages without rupturing and 40.3% of them display late elongation defects and stop developing without hatching (Table 2, line 4). These results show that *pix-1(gk416)* suppresses both the expressivity – the embryos arresting at 1.2–2 fold stages without rupturing – and the penetrance of *mel-11* early elongation defects at 25.5°C. Considering *mel-11(it26)* to be null at 25.5°C [19], these data suggest that *pix-1* functions in parallel with *mel-11* during early elongation. The aggravation of *pix-1* associated late elongation defects in a *mel-11(it26)* background is somewhat intriguing and provides the first evidence suggesting an involvement of *mel-11* in late elongation, in parallel with *pix-1*.

**Table 2:** Genetic interactions of *pix-1* and *pak-1* with *mel-11* and *let-502* mutants.

		18°C			25.5°C		
	Genotype	early elongation arrest (%)	Lva (%)	n	early elongation arrest (%)	Lva (%)	n
1	*wt*	0	0	531	0	0	323
2	*pix-1(gk416)*	0	5	420	0	8,5*	1190
3	*mel-11(it26)*	98$	0	508	100$	0	65
4	*mel-11(it26); pix-1(gk416)*	0	3	100	9,7$$	40,3***	62
5	*let-502(sb118ts)*	0	0	645	7,9	91,2**	353
6	*let-502(sb118ts); pix-1(gk416)*	18,1	2,5	764	89	11**	373
7	*mel-11(it26); let-502(sb118ts)*	96	0	905	24	0	487
8	*mel-11(it26); let-502(sb118ts); pix-1(gk416)*	29,8	2,2	359	43,1	31*	297
9	*pak-1(ok448)*	0	12	1714	0	25,8*	256
10	*mel-11(it26); pak-1(ok448)*	98	0	763	100	0	931
11	*let-502(sb118ts); pak-1(ok448)*	1	90	2417	100	0	2003
12	*mel-11(it26); let-502(sb118ts); pak-1(ok448)*	100	0	1066	100	0	891

Early elongation arrest include embryos arresting between comma and 1.75-fold with or without rupturing.

* arrested larvae present a *pix-1(gk416)* specific morphology (Figure 1A).

** arrested larvae present a *let-502 (sb118ts)* specific morphology (Figure 1A).

*** late elongation arrest without hatching.

$ all arrested embryos rupture.

$$ 0% rupture.

**Figure 3:**
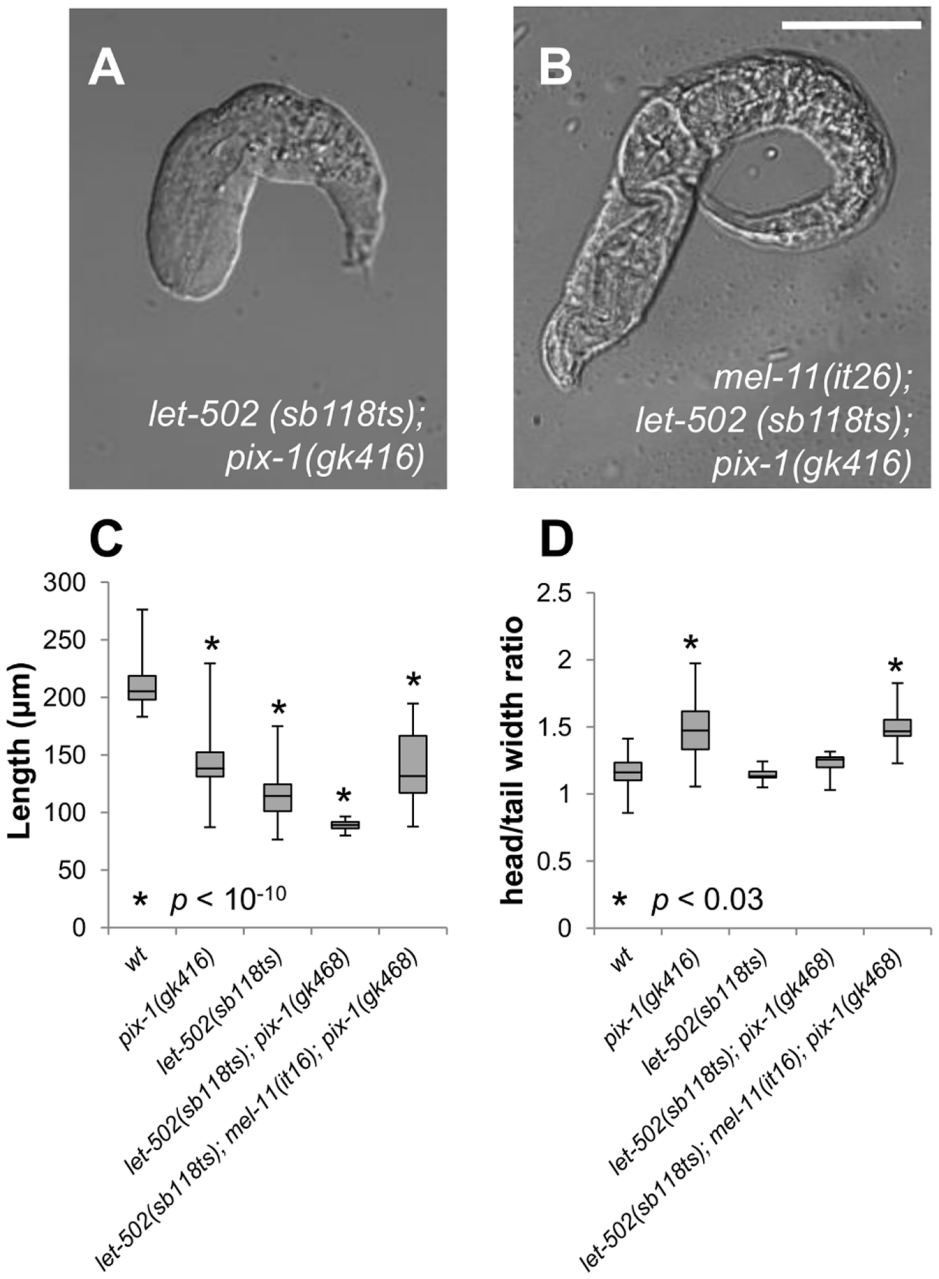
*pix-1(gk416)* controls early elongation in parallel with *mel-11/let-502*. A) Morphology of *let-502 (sb118ts); pix-1(gk416)* and B) *mel-11(it26)*; *let-502 (sb118ts); pix-1(gk416)* arrested larvae grown at 25.5°C. C) Distribution of sizes of arrested larvae in mutants' populations, at 25.5°C. Distribution of wild-type animals (*wt*) has been established using N2 L1 larvae synchronized by starvation after hypochlorite treatment. D) Distribution of ratio between the head and tail width of arrested larvae in mutant populations. The box-plot represents the min, max, 25^th^, 50^th^ (median) and 75^th^ percentiles of the population. Student's T-test *p-values* are indicated.

We then assessed whether *pix-1(gk416)* and *pak-1(ok448)* interacts with *let-502*(*sb118ts)* at 25.5°C. As previously shown, few *let-502*(*sb118ts)* embryos arrest between the 1.2- and 2-fold stage at restrictive temperature and the vast majority of the animals hatch as non-elongated larvae (Table 2, line 5, Figure 1A) [10]. 89% and 100% of *let-502(sb118ts)* embryos arrest between 1.2- and 2-fold stages in *pix-1(gk416)* and *pak-1(ok448)* backgrounds, respectively at 25.5°C (Table 2, line 6 and 11). In addition, *let-502(sb118ts); pix-1(gk416)* hatched larvae arrested with more severe elongation defects than *let-502(sb118ts)* animals (Table 2, line 6; Figure 3A and C), while their head/tail width ratio is not significantly different than *wt* as observed for *let-502(sb118ts)* arrested larvae (Figure 3D). These results suggest that *pix-1* functions in parallel with *let-502* to control elongation, as previously shown for *pak-1* [3], and that the loss of *let-502* function suppress the higher H/T ratio observed in *pix-1* mutant arrested larvae when compared to *wt*. Interestingly, the aggravation of *let-502* defects by *pix-1(gk416)* appears to be weaker than that observed for *pak-1(ok448)*, suggesting that *pix-1* is redundant with a yet unidentified gene in parallel with *let-502*.

To assess if *pix-1* functions in parallel with the *let-502/mel-11* pathway, we generated *mel-11(it26); let-502(sb118ts); pix-1(gk416)* triple mutants. At 18°C, *mel-11(it26); let-502(sb118ts)* display similar elongation defects to *mel-11(it26)* single mutant embryos due to the thermosensitive nature of *let-502(sb118ts)* (e.g. *wt* phenotype at 18°C; Table 2, compare lines 3 and 7). At non-restrictive temperature, a reduced number of *mel-11(it26); let-502(sb118ts); pix-1(gk416)* animals arrest during early elongation when compared to *mel-11(it26); let-502(sb118ts)* (Table 2, compare lines 7 and 8). This is consistent with our data showing suppression of *mel-11(it26)* early elongation defects by *pix-1(gk416)* (Table 2 line 4). At 25.5°C, *mel-11(it26); let-502(sb118ts)* are viable and embryos display 24% early elongation arrest (Table 2 line 7). At restrictive temperature, elongation defects are aggravated in *mel-11(it26); let-502(sb118ts); pix-1(gk416)* triple mutants in comparison to *mel-11(it26); let-502(sb118ts)* double mutants (Table 2, compare lines 7 and 8): 43.1% embryos arrested during early elongation (compared to 24% in *mel-11(it26); let-502(sb118ts)*), and 31% of animals hatched as non-elongated larvae presenting *pix-1* mutant-associated morphology (compared to 0% in *mel-11(it26); let-502(sb118ts)*; Figure 3B and C). Interestingly, *mel-11(it26); let-502(sb118ts); pix-1(gk416)* arrested larvae present a H/T width ratio significantly higher than *wt* and similar to that observed in *pix-1(gk416)* arrested larvae (Figure 3D). This suggests that in *mel-11(it26)* background, loss of *let-502* function is not anymore able to suppress *pix-1*-induced H/T ratio defect.

As previously shown, 100% of *mel-11(it26); let-502(sb118ts); pak-1(ok448*) embryos failed to hatch and displayed early elongation arrest (Table 2, line 12) [3]. These results suggest that *pix-1* functions in parallel with the *let-502/mel-11* pathway during early elongation, as shown for *pak-1*. However, the increased phenotypic severity observed in *pak-1 vs. pix-1* mutants suggests that *pix-1* functions redundantly with a gene or a group of genes in parallel with *mel-11/let-502* during early elongation. The different relationship observed between *mel-11* and *pix-1* or *pak-1* mutants also suggests that *pix-1* and *pak-1* have independent functions during early elongation. Whether this is the case during late elongation, could not be ascertained from our data.

### PIX-1::GFP is homogeneously distributed in the cytoplasm and at cell periphery of hypodermal cells

**Figure 4:**
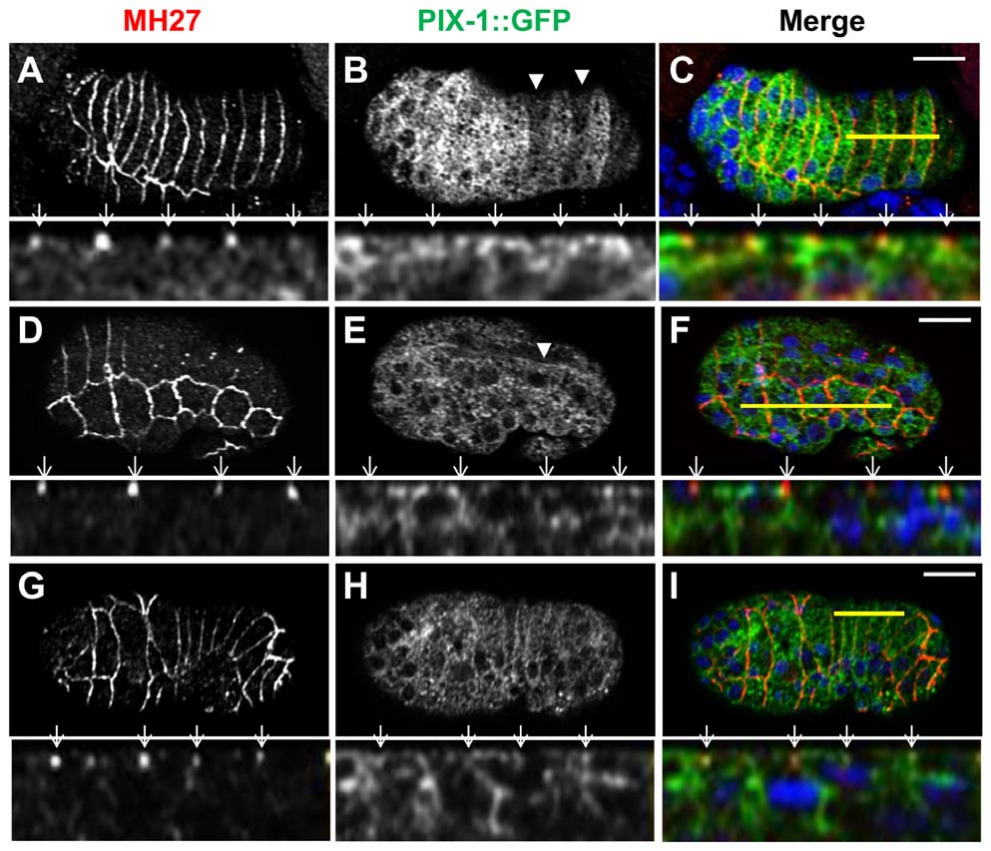
PIX-1 is homogeneously distributed in the cytoplasm and at the cell periphery of hypodermal cells. A–I) Immunostaining of *pix-1(gk416); sajEx1[pix-1p::pix-1::gfp]* expressing embryos with MH27 antibodies (A, D, G and red in merge panel C, F, I) and anti-GFP antibodies (B, E, H and green in merge panel C, F, I). Lower panel of each view correspond to orthogonal views of embryos Z-sectioning. Position of Z-sectioning is indicated in upper panel by a yellow line in dorsal (A–C) and lateral (D–F) and ventral (G–I) hypodermis. In orthogonal views, arrows point to adherens junctions which partially colocalize with PIX-1::GFP immunostaining. Arrowheads show the decrease in PIX::GFP expression every other cell in the dorsal-posterior hypodermis (at comma stage) in picture B (upper panel); Arrow-head indicates dorsal trans-epithelial attachment structures (TEA) in picture E (upper panel). Scale bars: 10 µm.

**Figure 5:**
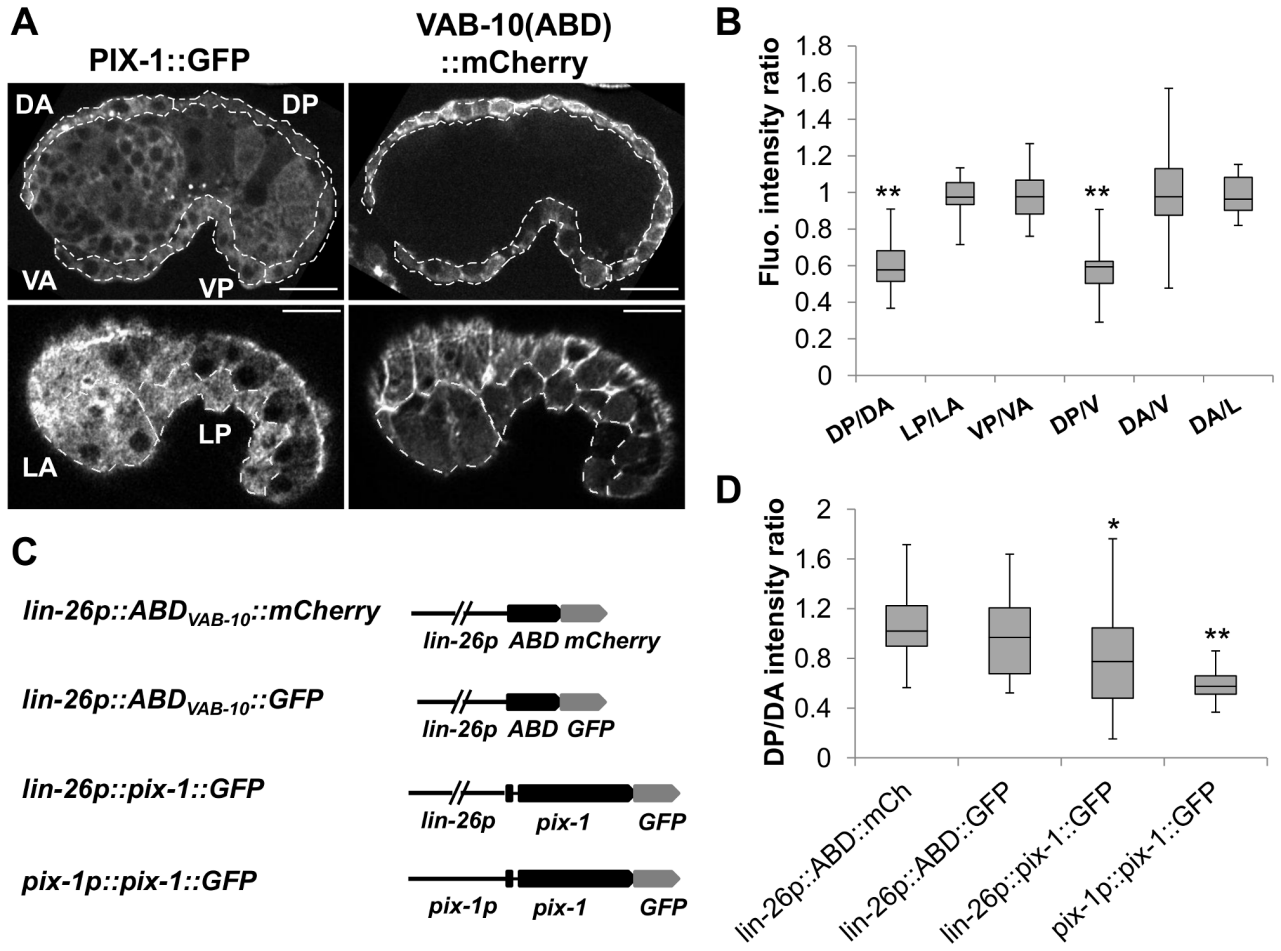
PIX-1::GFP is differentially expressed in hypodermal cells during elongation. A) Confocal lateral projections of *pix-1(gk416); sajEx1[pix-1p::pix-1::GFP]; mcIs40 [lin-26p::ABDvab-10::mcherry + myo-2p::gfp]* embryos. Dorsal-anterior (DA, upper panel), dorsal-posterior (DP, upper panel), ventral-anterior (VA, upper panel), ventral-posterior (VP, upper panel), lateral-anterior (LA, lower panel) and lateral-posterior (LP, lower panel) hypodermis are surrounded by dashed line and have been identified using *lin-26p::vab-10(ABD)::MCHERRY* hypodermal markers. B) Distributions of the dorsal-posterior/dorsal-anterior (DP/DA), lateral-posterior/lateral-anterior (LP/LA), ventral-posterior/ventral-anterior (VP/VA), dorsal-posterior/ventral (DP/V); dorsal-anterior/ventral (DA/V), dorsal-anterior/lateral (DA/L) rates of fluorescence intensity measured in *pix-1(gk416)*; *sajEx1[pix-1p::pix-1::GFP; rol-6]; mcIs40 [lin-26p::ABDvab-10::mcherry + myo-2p::gfp]* embryos between comma and 1.75-fold stages (n = 26 embryos). Similar results were also obtained in *pix-1(gk416)*; *sajIs1[pix-1p::pix-1::GFP; unc-119^R^]*; *mcIs40 [lin-26p::ABDvab-10::mcherry + myo-2p::gfp]*. The box-plots represent the min, max, 25^th^, 50^th^ (median) and 75^th^ percentiles of the populations. ** T-test comparing ratios to 1 *p*<0.01. C) Schematic representation of *pix-1::GFP* and ABD_VAB-10_ (control) constructs used to measure the DP/DA intensity ratio reported in panel D. DP/DA of animals carrying *mcIs40* (*lin-26p::ABD::mCh* expressing), *mcIs50* (*lin-26p::ABD::GFP* expressing), *sajIs2* (*lin-26p::pix-1::GFP* expressing) or *sajEx1* (*pix-1p::pix-1::GFP* expressing). ratios** T-test comparing DP/DA ratios measured on *pix-1::GFP* expressing embryos to ratio measured in ABD_VAB-10_ expressing transgenics, *p*<0.01. The box-plots represent the min, max, 25^th^, 50^th^ (median) and 75^th^ percentiles of populations.

*pix-1* was previously shown to be involved in mechanotransduction signaling in ventral and dorsal hypodermal cells upon muscle contraction during late elongation events [4]. The localisation of PIX-1 at the TEA in dorsal and ventral hypodermal cells supports this function [4]. Our data suggest that *pix-1* also controls early elongation events. These events are thought to be directed by the contraction of filamentous actin bundles (FBs) at the cell periphery and at the most apical part of the lateral hypodermal cells [3]. To better understand the function of PIX-1 during early elongation, we characterized its subcellular localization in hypodermal cells during early elongation. To do so, we examined the localisation of PIX-1::GFP expressed under the control of the *pix-1* endogenous promoter in *pix-1(gk416)* animals immuno-stained with MH27 antibodies (staining of hypodermal adherens junctions; Figure 4) or expressing the filamentous actin-binding probe VAB-10_ABD_::mCherry in hypodermal cells (Figure S4). Confocal microscopy analysis revealed that during early elongation, PIX-1::GFP is expressed in the dorsal (Figure 4A-C, Figure S4A–F), lateral (Figure 4D–F) and ventral hypodermal cells (Figure 4G–I, Figure S4D–F). Throughout early elongation, PIX-1::GFP is located at the TEA in dorsal and ventral hypodermal cells, as previously reported (Figure 4 E, arrow-head) [4] and is also homogeneously distributed in the cytoplasm and at the cell periphery of all expressing cells (Figure 4, Figure S4). Interestingly, at the comma stage, PIX-1::GFP expression appears to be reduced in several posterior dorsal hypodermal cells (Figure 4 B, arrowhead). Concomitant with the fusion of these cells, around the 1.2-fold stage, the expression of PIX-1::GFP appears to be reduced in the fused cells (Figure 5A).

### High expression of *pix-1* in dorsal posterior hypodermis is detrimental for early elongation

Considering that *pix-1* controls reduction of the head but not the tail width of the embryos during early elongation, we were intrigued by the reduction of the expression of PIX-1::GFP in the dorsal-posterior hypodermal cells in the transgenic animals. We then quantified PIX-1::GFP expression in dorsal-anterior (DA), dorsal-posterior (DP), ventral-anterior (VA), ventral-posterior (VP), lateral-anterior (LA) and lateral-posterior (LP) hypodermal cells (Figure 5 A). This study revealed a significant lower ratio of DP/DA and DP/V expression when compared to LP/LA, VP/VA, DA/V and DA/L ratios, which were not significantly different than 1 (Figure 5 B). Similar data were obtained using two independent transgenic lines expressing translational fusions of PIX-1::GFP under the control of the *pix-1* endogenous promoter, one transgenic line carrying an extrachromosomal array (*pix-1(gk416);sajEx1*) and one stable transgenic line carrying an integrated array (*unc-119(ed3);pix-1(gk416);sajIs1* see Methods). We also found that all measured ratios were constant throughout early elongation (Figure S5).

**Figure 6:**
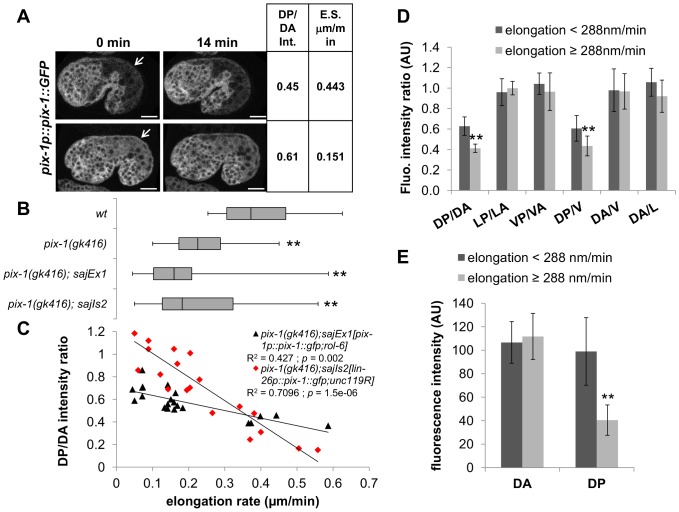
High expression of PIX-1::GFP in dorsal posterior hypodermis is detrimental for elongation rate of embryos. A) Confocal projections of *pix-1(gk416); sajEx1[pix-1p::pix-1::GFP, rol-6]* at t = 0 min and t = 14 min. The DP/DA fluorescence intensity ratio and elongation rate (in μm/min) are indicated. Arrows indicate the dorsal-posterior hypodermis. Scale bar: 10 µm. B) Distribution of elongation rate in μm/min of *wild-type (wt)*, *pix-1(gk416)*, and *pix-1(gk416)* animals carrying *sajEx1[pix-1p::pix-1::GFP, rol-6]* or *sajIs2[lin-26p::pix-1::GFP, unc-119^R^]* during early elongation. Elongation rate was measured from 4-dimensional recording of embryonic development between comma and the end of early elongation upon DIC illumination. Box-plots represent the min, max, 25^th^, 50^th^ (median) and 75^th^ percentile of the populations. ** T-test *p*<0.01 vs *wt*. C) Scatter plot representing the relationship between the dorsal-posterior/dorsal-anterior (DP/DA) intensity ratio of PIX-1::GFP and the elongation rate in μm/min during early elongation of *pix-1(gk416)* embryos carrying *sajEx1[pix-1p::pix-1::GFP, rol-6]* or *sajIs2[lin-26p::pix-1::GFP, unc-119^R^]* (n = 20 for each line). The spearman correlation (R2) between the elongation rate and the DP/DA ratio are indicated, as well as the *p-values* rejecting the null hypothesis being that the two values are not significantly correlated. Similar results were obtained for *pix-1(gk416)* animals carrying *sajIs1* and *sajIs3* (see methods). D) DP/DA, lateral-posterior/lateral-anterior (LP/LA), ventral-posterior/ventral-anterior (VP/VA), dorsal-posterior/ventral (DP/V), dorsal-anterior/ventral (DA/V) and dorsal-anterior/lateral (DA/L) fluorescence intensity ratio measured for *pix-1(gk416); sajEx1[pix-1p::pix-1::GFP, rol-6]* embryos elongating at a *wt*-rate (elongation≥288nm/min) or elongating slower (elongation<288nm/min) during early elongation. Bar correspond to the mean and error bars to the standard deviation. ** T-test p-value<0.01. E) PIX-1::GFP fluorescence intensity (AU) was measured in DA and DP hypodermal cells of embryos elongating at a *wt*-rate (elongation≥288nm/min) or elongating slower (elongation<288nm/min) during early elongation (see methods). ** T-test p-value<0.01.

We then assessed whether the *sajEx1[pix-1p::pix-1::GFP,rol-6]* transgene could rescue elongation defects in *pix-1(gk416)* animals. We found that the transgene significantly rescued the larval arrest phenotype (Lva) of *pix-1(gk416)* from 8.5% (N = 1216) to 2.0% (N = 917) (Table 1; T-test, *p-value* = 0.03). Using time-lapse microscopy, we also measured the elongation rate of wild-type (*wt*), *pix-1(gk416)* and *pix-1(gk416);sajEx1[pix-1p::pix-1::GFP, rol-6]* embryos and found that the transgene did not significantly rescue the elongation rate of mutant animals (Figure 6 B). This suggests that while the expression of PIX-1::GFP fusion protein is sufficient to support PIX-1 function during early elongation (rescuing Lva), it was not as efficient as the endogenous protein.

We then observed the elongation rate of embryos at the single animal level and attempted to see if there was any correlation between a given expression pattern of the transgene and the ability of the transgene to efficiently rescue the mutant elongation rate defect. We found that transgenic embryos with similar elongation rates to *wt* embryos (elongation≥288 nm/min, Figure 6D) had DP/DA and DP/V ratios of PIX-1::GFP intensity that were significantly lower than embryos that elongated slower (elongation<288 nm/min, *p-value*<0.01; Figure 6D). No significant difference was observed for the LP/LA, VP/VA DA/V and DA/L ratios between the two populations of embryos (Figure 6D). These data show that PIX-1::GFP expression was significantly reduced in dorsal-posterior cells when compared to dorsal-anterior and ventral cells in rescuing animals. Such reduction of PIX-1::GFP expression in dorsal-posterior cells was not observed in non-rescuing animals. Importantly, animals presenting a DP/DA ratio higher than 0.5, elongated almost 3 times slower than *wt* animals (Figure 6A). Overall, the speed of early elongation appeared to be negatively correlated to the DP/DA ratio of PIX-1::GFP expression (Spearman correlation coefficient R^2^>0.427; *p-value*<0.002; Figure 6C), but not to any other PIX-1::GFP intensity ratio measured in hypodermal cells (Figure S6). Similar results were obtained using two independent *pixp::pix-1::GFP* expressing transgenic lines, one carrying an extrachromosomal array (*pix-1(gk416);sajEx1*), and a stable transgenic line carrying an integrated array (*unc-119(ed3);pix-1(gk416);sajIs1)*. These data suggest that transgenic animals expressing PIX-1::GFP homogeneously in dorsal-anterior, lateral and ventral hypodermal cells, but two times less in dorsal-posterior cells, elongate at a *wt-*rate during early elongation. However, animals expressing the transgene in dorsal-posterior cells at a similar/higher level when compared to other cells elongated significantly slower.

PIX-1::GFP is expressed in cells other than the hypodermis, and we cannot exclude the possibility that differential expression of PIX-1::GFP in other cells may affect the rescuing ability of the transgene. To test this possibility, we generated transgenic animals expressing PIX-1::GFP under the control of the hypodermal specific promoter, *lin-26p*. This promoter is thought to drive the expression of coding sequences only in hypodermal cells and in a homogenous manner [3]. We confirmed this later assumption through measurements of the DP/DA fluorescence intensity ratios in *lin26p::vab-10(ABD)::GFP* and *lin-26p::vab-10(ABD)::mCherry* transgenic animals carrying integrated arrays expressing actin-binding fluorescent probes in all hypodermal cells under the control of *lin-26p* [3]. We showed that these ratio were not significantly different than 1 (Figure 5 C and D).

As observed with *pix-1p::pix-1::GFP* expressing animals, transgenic animals carrying an integrated array expressing PIX-1::GFP under the control of *lin-26p* segregated into two populations. The embryos of the first population displayed an elongation rate similar to *wt* (elongation rate≥288 nm/min, Figure S7) and had DP/DA and DP/L intensity ratios that were significantly lower than the second population of embryos that elongated slower (elongation rate<288 nm/min, Figure S7). As shown for transgenic animals expressing *pix-1p::pix-1::GFP*, all animals expressing PIX-1::GFP under the control of *lin-26p* express similar levels of the transgene in DA, L and V cells (Figure S7). These data support our hypothesis that a DP/DA PIX-1::GFP expression ratio higher than 0.5 may hinder early elongation.

To determine if the lower DP/DA, DP/L and DP/V intensity ratios observed in embryos with a *wt*-like elongation rate were due to a reduced expression of PIX-1::GFP in DP cells or to an increased expression of PIX-1::GFP in DA, L and V cells, we measured the fluorescence intensity of PIX-1::GFP in DA and DP cells in *lin-26p::pix-1::GFP* expressing animals. We found that the PIX-1::GFP fluorescence intensity was not significantly different in the DA cells of embryos developing at a *wt*-rate when compared to those developing at slower rates (Figure 6 E). However, PIX-1::GFP fluorescence intensity appeared to be significantly lower in the DP hypodermal cells in the subpopulation of embryos that elongated faster. This phenomenon was observed in two independent stable transgenic lines. These data suggest that the DP/DA ratios in embryos that elongated at a *wt*-rate was not due to an increased expression of PIX-1::GFP in the DA cells, but to decreased PIX-1::GFP expression in the DP cells.

Altogether, these data suggest that homogenous expression of PIX-1::GFP in dorsal-anterior, lateral and ventral hypodermal cells, and decreased expression in dorsal-posterior cells significantly rescued the elongation defects observed in the *pix-1(gk416)* animals. They also suggest that expression of the transgene in dorsal-posterior cells above a threshold corresponding to half the expression in the other hypodermal cells decreases the efficiency of early elongation. This suggests that *pix-1* may be submitted to a tight control of its expression level in hypodermal cells, particularly to decrease its level in the dorsal-posterior cells.

## Discussion

Embryonic elongation transforms the ovoid embryo into the long, thin vermiform nematode. This morphogenetic process occurs in two phases. The early elongation is driven by the contraction of Filamentous actin Bundles (FBs) in hypodermal cells. The late elongation is driven by a mechanotransduction pathway in the dorsal and ventral hypodermis resulting from contraction of the underlying muscle cells.

Two parallel pathways control early elongation. One pathway involves two kinases: the RHO-1/RHOA effector LET-502/ROCK and the ortholog of the CDC-42-effector human myotonic dystrophy kinase MRCK-1/MRCK [8], [10]. Both kinases are thought to inhibit the function of MEL-11/PP-1M thus permitting phorphorylation of myosin-light chain (MLC-4/MLC) and contraction of FBs. The second pathway involves the CDC42/RAC-effector PAK-1, the PP2C phosphatase FEM-2/POPX2 and a RHO/RAC GTP nucleotide-exchange factor (GEF) UNC-73/TRIO. The function of these two later genes in the regulation of MLC-4/MLC phosphorylation and/or PAK-1 function remains unknown [3], [8], [10], [16]. Downstream of the two parallel pathways LET-502/ROCK and PAK-1 are thought to phosphorylate MLC-4/MLC in the hypodermal cells and to control contraction of FBs. This contraction is assumed to be high in the lateral and low in the ventral and dorsal hypodermal cells during early elongation [11].

In this study, we show that the CDC42/RAC-GEF *pix-1* controls early elongation in parallel with the *mel-11/let-502* pathway. Our data suggest that *pix-1* controls this process as part of the *pak-1* pathway in parallel with a gene (or a group of genes) that remains to be identified. *pix-1* may also function independently from *pak-1*. Our data suggest that PIX-1/PAK-1 and LET-502/ROCK have different function along the antero-posterior axis of the embryo during early elongation: PIX-1, PAK-1 and LET-502/ROCK appear to control the constriction of the head while only LET-502/ROCK controls the constriction of the tail of the elongating embryo. Our study also revealed that PIX-1::GFP fusion protein is homogeneously distributed in the cytoplasm and at the cell periphery of hypodermis during early elongation. We showed that this fusion protein rescues the reduced elongation rate observed *pix-1(gk416)* in a subset of transgenic animals expressing the transgene homogenously in dorsal-anterior, lateral and ventral cells and at a lower level in dorsal-posterior cells. Our results also suggest that PIX-1 expression above a certain threshold in dorsal-posterior hypodermal cells is detrimental for early elongation.

### *pix-1* functions with unidentified genes in parallel with *mel-11/let-502*

**Figure 7:**
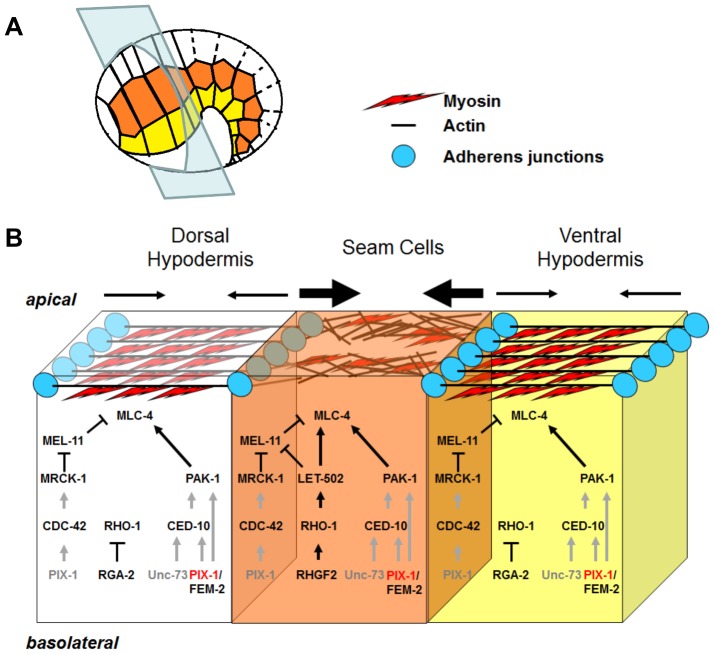
Model for signaling pathways controlling embryonic elongation. A) Schematic representation of an embryo during early elongation. Anterior is at the left and dorsal side on the top. Dorsal (white), lateral (orange) and ventral (yellow) hypodermal cells are represented. The blue plan indicates the location of the transversal sectioning of the hypodermal cells represented in panel B. B) Signaling pathways in the dorsal (white), lateral (orange) and ventral (yellow) hypodermis in the anterior part of the embryo during early elongation. In this model PIX-1 is expressed at similar level in all hypodermal cells of the anterior part of the embryo. While homogenous expression of PIX-1::GFP in these cells rescues elongation defects of *pix-1(gk416)*, we cannot exclude the possibility that *pix-1* may be required only in a subset of these cells. In PIX-1-expressing cells, PAK-1 is activated in a GTPase-dependant (through activation of CED-10 by PIX-1 and/or UNC-73) or in a GTPase-independent manner (by PIX-1 directly). LET-502 is activated only in seam cells through activation of RHO-1 by RHGF-2. PIX-1 may also activate MRCK-1 through CDC-42 upstream of MEL-11. Following this model, the contraction pressure applied on the actin cytoskeleton is similar in ventral and dorsal hypodermis and higher in seams cells. Arrows represent relative contraction forces within each cell.

Both *pix-1* and *pak-1* mutants have similar elongation phenotypes and both aggravate the elongation defects observed in *mel-11; let-502* double mutant suggesting that these two genes control together a subset of developmental mechanisms in parallel with *mel-11; let-502* during early elongation. However, *pix-1* mutant aggravates elongation defects associated with *mel-11; let-502* to a lesser extent than the *pak-1* mutant. This suggests that *pix-1* functions with other genes that act in parallel with *mel-11/let-502* pathway (Figure 7).

In both mammals and *C. elegans* βPIX/PIX-1 was shown to activate PAK-1 kinase activity in a GTPase-dependent (canonical) or GTPase-independent (non-canonical) manner [4], [17], [18]. M. Labouesse's laboratory (IGBMC, Illkirch, France) showed using GTPase pull-down assays that the level of activation of the Rho GTPase CED-10/RAC was significantly lower in *pix-1(gk416)* than in *wt* embryos, suggesting that PIX-1 regulates CED-10/RAC activity during embryonic development [4]. We can then hypothesize, that PIX-1 may activate PAK-1 in a canonical manner through CED-10/RAC during early elongation as shown during late elongation [4]. Supporting this hypothesis, CED-10/RAC was located at both cell junctions and at the TEA within hypodermal cells in elongating embryos [4], [20]. However, we cannot exclude the possibility that PIX-1 may also activate PAK-1 during early elongation in a GTPase-independant/non-canonical manner as shown during gonad morphogenesis [17], [18]. Interestingly, the RHO/RAC specific GEF *unc-73/*TRIO was also shown to control early elongation in parallel to *mel-11/let-502* [10]. The allele used in that study, *rh40*, consists of a missense mutation that eliminates its exchange activity towards CED-10/RAC, RAC-2/RAC and MIG-2/RHOG without affecting its activity towards RHO-1/RHOA [15], [21]. A UNC-73/TRIO - CED-10/RAC - PAK-1 pathway would then be an excellent candidate pathway controlling early elongation in parallel with a non-canonical PIX-1 – PAK-1 or a canonical PIX-1 - CED-10/RAC - PAK-1 pathway (Figure 7). Careful study will be required to test these hypotheses and to better understand the molecular mechanisms involving PIX-1 and controlling the activity of PAK-1 during early elongation.

### *pix-1* may control early elongation in a *pak-1*-dependent manner

While *pix-1* and *pak-1* may be part of the same pathway in parallel with *mel-11/let-502, mel-11(it26)*-inducing embryo rupturing is suppressed by *pix-1(gk416)* but not by *pak-1(ok448)* (Table 2). This intriguing genetic interaction between *pix-1* and *mel-11/*PP-1M during elongation is similar to that observed between *fem-2/*POPX2 and *mel-11*/PP-1M, another gene shown to be part of the *pak-1* pathway [16].

It was shown that the *fem-2* null allele induces weak larval arrest with non-elongated larvae similar to that observed in *pix-1* and *pak-1* mutants [10]. The *fem-2* mutant can also aggravate *mel-11*; *let-502* double mutants and suppress the *mel-11* rupturing phenotype. [10], [16]. This suggests that *pix-1*, and *fem-2/*POPX2 function together to control early elongation in parallel with *mel-11/let-502*. Interestingly, the PP2C-like serine/threonine phosphatases, POPX2/Protein Phosphatase 1F, the closest homolog of FEM-2 in mammals, was shown to interact with β-Pix (ortholog of PIX-1) and to dephosphorylate and inactivate PAK1 (ortholog of PAK-1*)* kinase activity in mammalian cells [22]. It was suggested that the β-PIX - POPX2 complex controls the activation/inactivation turnover of PAK1 in mammalian cells [22], [23]. Considering that these proteins are highly conserved between *C. elegans* and mammals, we hypothesize that PIX-1, FEM-2/POPX2 and PAK-1 may have functional relationships similar to their homologs in mammals. Following this hypothesis, PIX-1 and FEM-2/POPX2 may control the activation/inactivation turnover dynamics of PAK-1 during early elongation. Considering that *pix-1* and *fem-2* mutant have similar interactions with *mel-11* and *let-502* during early elongation, this hypothesis suggests that a reduced or a sustained activation of PAK-1 alters similarly early elongation process. While this hypothesis would explain the genetic data obtained with *pix-1*, *fem-2* and *pak-1* mutants during elongation, it still remains to be confirmed through a careful analysis of a possible relationship existing between PAK-1 activation/inactivation dynamics and the regulation of myosin contraction in *C. elegans* hypodermal cells during early elongation.

### *pix-1* may control early elongation in a *pak-1*-independent manner

Considering the molecular analysis of *mel-11(it26)* allele [19], we cannot exclude the possibility that this allele may not be completely null even at 25.5°C. We can then hypothesize that *pix-1* may function together with *pak-1* in parallel with *mel-11* and may also function upstream of *mel-11* independently of *pak-1*. This would constitute an alternative explanation for the suppression of *mel-11(it26)*-inducing rupturing by *pix-1* but not by *pak-1* allele. Interestingly, the kinase MRCK-1/MRCK, whose mammalian ortholog, is an effector of CDC-42 [24] was shown to control early elongation upstream of *mel-11* [3]. CDC-42 being expressed in hypodermal cells during early elongation [20], and being also a potential target of PIX-1 GEF activity, we can hypothesize that PIX-1 may activate MRCK-1 through CDC-42 upstream of MEL-11 and may also activate PAK-1 in parallel with *mel-11/let-502* (Figure 7).

### The *pix-1/pak-1* pathway mainly controls the constriction of the head of the embryos during early elongation

Observation of the head to tail morphology of the elongating embryo showed that the anterior part of the embryo at 1.2-fold stage is much wider than the posterior part. This suggests that contraction forces applied on the anterior part of the embryo is higher than in the posterior part at that stage. We showed that the mechanism leading to a faster reduction of the head width during early elongation is dependent on *pix-1*, *pak-1* and *let-502/*ROCK and that reduction of the tail width is dependent on *let-502/*ROCK but neither on *pix-1* nor on *pak-1*. This suggests that contraction forces controlled by *let-502/*ROCK are required for the embryo morphogenesis along the antero-posterior axis, while those controlled by the *pix-1/pak-1* pathway may mostly be required in the anterior part of the embryo. This also suggests that either the *pix-1/pak-1* pathway induces contractions mostly in the anterior part of the embryo, or this pathway is redundant with another pathway (in addition to *let-502/mel-11)* specifically involved in the control of contractions in the posterior part of the embryo.

Interestingly, we found that animal expressing PIX-1::GFP at a reduced level in the dorsal posterior cells present an elongation rate similar to *wt*. Higher expression of PIX-1::GFP in the dorsal-posterior cells is also shown to be detrimental for the elongation rate of embryos during early elongation. This suggests that the function of *pix-1* is required at a low level in the dorsal-posterior hypodermal cells to ensure optimal elongation rate. This supports the hypothesis that the *pix-1/pak-1* pathway may induce more contractions in the anterior part of the embryo than in the posterior part. However, this hypothesis will have to be tested through careful measurement of contraction forces induced by the *let-502/mel-11* and *pix-1/pak-1* pathways in individual sets of hypodermal cells during early elongation.

We also showed that loss of *let-502* function suppresses the increased head/tail width ratio observed in *pix-1* arrested larvae when compared to *wt* and that this suppression occurs only in embryos expressing an active *mel-11* (Figure 3D). These data support our observations that *let-502; pix-1* arrested larvae display *let-502* morphology, while *let-502; mel-11; pix-1* larvae display *pix-1* morphology (Table 2). To explain these results, we may hypothesize that loss of *let-502* may induce a severe reduction of contraction forces in hypodermal cells due to the activation of *mel-11*. This activated *mel-11* may then strongly suppress the contraction forces induced by the *pix-1/pak-1* pathway and consequently the difference of contraction forces applied on the head and tail during early elongation. According to this hypothesis, in absence of both *let-502* and *mel-11*, deletion of *pix-1* function is associated with the arrest of larvae displaying a similar increase of H/T width ratio than observed in single *pix-1(gk416)* mutants. These data confirm that MEL-11 function antagonizes both contraction forces induced by LET-502 and those induced by the PIX-1/PAK-1 pathway (Figure 7) [3], [14].

In summary, our study demonstrates a function for *pix-1* during early elongation within the *pix-1/pak-1* pathway in parallel with *mel-11/let-502*. At that stage, *let-502* may drive the contraction forces leading the reduction of the embryo circumference along the antero-posterior axis, while the *pix-1/pak-1* pathway may mainly control the contraction forces applied on the anterior part of the embryo.

## Methods

### Strains and Culture Methods

Control N2 and other animals were maintained in standard conditions at 20°C [23]. Worm strains carrying the following mutations and markers: *pix-1(gk416) X, pix-1(ok982) X, pak-1(ok448) X, mcIs40 [lin-26p::ABDvab-10::mcherry + myo-2p::gfp]* and *mcIs50 [lin-26p::ABDvab-10::gfp + myo-2p::gfp],* were obtained from the *Caenorhabditis Genetic Center* (CGC). Mutant strains were backcrossed at least 3 times against wild-type (*wt*) animals prior to analysis. Strains carrying *let-502(sb118) I, mel-11(it26) unc-4(e120)/mnC1 II* and *mel-11(it26) unc-4(e120) II; let-502(sb118) I,* were kindly provided by Dr Paul Mains (University of Calgary, Calgary, Canada). *mel-11(it26) unc-4(e120) II; let-502(sb118) I* were maintained at 25.5°C. *mel-11(it26) unc-4(e120) II; let-502(sb118) I; pix-1(gk416) X* and *mel-11(it26) unc-4(e120) II; let-502(sb118) I; pak-1(ok448) X* were generated after crossing *mel-11(it26) unc-4(e120) II; let-502(sb118) I* hermaphrodites with *pix-1(gk416) X* or *pak-1(ok448) X* males. Genotyping of F2 progeny was done through isolation of Unc F2 (*mel-11(it26) unc-4 (e120) II homozygotes)* at 15°C. Mutations in *pix-1* and *pak-1* genes were identified using Polymerase chain Reaction (PCR) and *let-502(sb118) I* homozygotes were identified through scoring of embryonic lethality (Emb) and larval arrest (Lva) phenotypes of populations grown at 18°C and 25.5°C.

### Generation of Transgenic animals

*pix(gk416); sajEx1[pix-1p::pix-1::GFP;rol-6(su1006)]* animals were generated by injection. Translational PIX-1::GFP fusion construct (*pix-1p::pix-1::gfp*) was obtained from Dr Chen HJ's laboratory (University of California Davis, Davis, California, USA) [17] and injected at 15 ng/μl with pRF4 (containing *rol-6(su1006)* at 50 ng/μl) in *pix(gk416)* animals. Rol transgenic animals were isolated and expression of PIX-1::GFP was assessed by fluorescent microscopy. *unc-119(ed3); pix-1(gk416); sajIs1[pix-1p::pix-1::gfp; unc-119^R^]* was generated using biolistic bombardment. To do so, constructs were generated through amplification of the *pix-1* promoter (1.6 kb upstream of the initiation codon), amplification using reverse-transcription and PCR using thermoscript II (life technologies) kit of *pix-1* cDNA (from the ATG the codon in 5′of the endogenous stop codon, including the intron between exon 1 and 2). Both DNA fragments were inserted using gateway recombination in pDONRP4P1R and pDONR201 respectively and recombined together in pMB14. This construct was integrated by biolistic bombardment in *unc-119(ed3); pix-1(gk416)* strain, using a PDS-1000/He system with the Hepta adaptor (Bio-Rad) as previously reported [24]. *unc-119(ed3); pix-1(gk416); sajIs2[lin-26p::pix-1::GFP]* and *unc-119(ed3); pix-1(gk416); sajIs3[lin-26p::pix-1::GFP]* were independent, stable transgenic lines generated also using biolostic bombardment. It contains 5kb of the *lin-26* promoter *(lin-26p)*, the *pix-1* coding sequence including the intron between exon 1 and 2, and the GFP coding sequence in pMB14 vector. This construct was also integrated by biolistic bombardment in *unc-119(ed3); pix-1(gk416)* strain, using a PDS-1000/He system with the Hepta adaptor (Bio-Rad).

Transgenic animals expressing PIX-1::GFP together VAB-10(ABD)::mCherry were obtained through crossing *pix(gk416); sajEx1*, *unc-119(ed3); pix-1(gk416); sajIs1*, *unc-119(ed3); pix-1(gk416); sajIs2* and *unc-119(ed3); pix-1(gk416); sajIs3* hermaphrodites with *mcIs40[lin-26p::ABDvab-10::mcherry+myo-2p::gfp]* males. Stable lines expressing GFP, mCherry transgenes and carrying *pix-1(gk416)* allele were isolated from the F2 progeny.

### Phenotyping mutant animals and 4-dimensional microscopy

To score Emb and Lva phenotypes, worms were synchronized by hypochlorite treatment. After synchronisation 10–20 worms were deposited on NGM agar with OP50 as a source of food Worms were allowed to lay eggs at 18°C or 25.5°C for 4 to 5 hours and were washed off the plate with M9 medium. After 24 and 48 hours dead eggs and arrested L1 larvae were counted and observed at high magnification. The stage of embryonic arrest was confirmed in mutant animals using four-dimension microscopy. Embryos dissected from adult hermaphrodites were mounted on 3% agarose pads in M9 buffer and coverslips were sealed with drawing gum (pébéo). Elongation was recorded using 4-dimensional microscopy (3D and time), which recorded a Z-stack every 2 minutes during 10 hours at 23–24°C using a Leica DM5500 microscope equipped with a 63X oil immersion objective upon differential interference contrast illumination (DIC). Images were captured using the Leica LAS AF imaging software. These recording were used to measure the duration of early elongation for at least 20 eggs from 1.2- to the end of early elongation – identified as the moment when body-wall muscles start contracting, the length of the embryos at the end of early elongation, the width of the head (measured at equidistance from the tip of the nose to the pharynx-intestinal valve), the width of the tail (measured at equidistance from the pharynx-intestinal valve to the distal extremity of hyp10) at 1.2-fold and at the end of early elongation in the different mutant animals. These measurements were done using Leica LAS AF6000 imaging analysis tools. The reproducibility of these measurements was tested as detailed in the supplementary Figure 3. Length and head/tail width measurements were also done on mutant arrested larvae and wild-type (*wt*) L1 arrested by starvation after hypochlorite treatment. The head/tail ratio was calculated as the ratio of the head width with the tail width in μm per animal. The head (and tail) width reduction ratio was calculated as the ratio of the head width (or tail width) at 1.2-fold stage over the head width (or tail width) at the end of early elongation. Statistical significance was calculated using unpaired Student's T-test.

### Immunostaining of embryos

For indirect immunofluorescence, embryos were fixed using 3% paraformaldehyde at room temperature for 10 minutes. Following washes with PBS (137 mM NaCl, 2.7 mM KCl, 4.3 mM Na_2_HPO_4_, 1.47 mM KH_2_PO_4_ Adjust to a final pH of 7.4.), embryos were incubated 10 minutes in cold methanol, and extensively washed with PBS. Fixed embryos were incubated overnight at 4°C with appropriate dilutions of primary antibodies in culture media (4X eggs salt, 0.5% Hepes 1M, 5% goat serum). Mouse anti-MH-27 antibodies (The Developmental Studies Hybridoma Bank, University of Iowa) were used at 1∶10 dilution, rabbit anti-GFP antibodies were used at 1∶500 (Invitrogen). After three washes with culture media (4X egg salt, 0.5% HEPES 1M, goat serum 5%), embryos were incubated at room temperature for 1 h with 1∶200 dilution of either anti-mouse IgG conjugated to TRITC or anti-rabbit IgG conjugated to FITC (Jackson ImmunoResearch) in 1X-PBS. Embryos were washed three times with culture media and incubated with 100 ng/ml of DAPI for 1 minute at room temperature, washed with water, resuspended in 40 µl of Mowiol (sigma Aldrich) and mounted on slides.

### Confocal fluorescence microscopy

The expression pattern of PIX-1::GFP in living animals was observed using a Nikon A1R confocal microscope with 100X oil CFI NA 1.45 Plan Apochromat λ objective. All images were captured with a pinhole size of 59.1 µm, with a calibration of 0.12 µm/pixel (radial resolution of 0.20 µm) and a Z-step of 0.15 µm. Images were captures using NIS-element software (Nikon). Deconvolution was done using Autoquant 3X, 3D deconvolution software. Orthogonal views, and fluorescence quantifications were generated using ImageJ software. For fluorescence quantification, individual Z-steps were extracted from Z-stacks recording of elongating embryos. DP, DA, LP, LA, VP, VA hypodermal cells were selected using ImageJ ‘polygon selections’ tool. Nuclei of cells in these areas were also selected using the same tool. The Raw intensities of selected areas were measured using area and gray value function. Raw Intensities of nuclei were subtracted to raw intensities of selected hypodermal sections. The pixel mean value was then calculated dividing this adjusted raw intensity by the area size selected excluding also size of nuclei. Intensity ratios and raw intensities were calculated and used using the resulting value. Comparison of raw intensities between different animals was done on image captured within a week, to limit the impact of laser fluctuation. This fluctuation was controlled through comparison of intensities obtained for the same sample at different days. Fluorescence intensity was compared on images captured using the same laser power and adjusted photomultiplier tube (PMT). Statistical significance of quantification was assessed using the unpaired student T-test. Spearman correlation coefficients and statistical tests for significance of correlation between two quantitative variables were calculated using spearman cor.test function in R (Bioconductor).

## Supporting Information

Figure S1 **Schematic representation of human (Hs) and** ***C. elegans*** **(Ce) PIX and PAKs.** Modular structure has been identified using the SMART tool (www.smart.org) or by alignment of consensus sequence using CLUSTALW. Binding-domains for protein partners reported in the literature are indicated. Proteins coded by *C. elegans* mutant alleles are indicated. * indicate the location of translation arrest. CH: calponin homology domain; SH3: src homology domain; DH: dbl homology domain; PH: Pleckstrin homology domain; PR: Proline Rich sequence, GBD: GIT-binding domain, CC: coil-coiled domain, ZB: PDZ binding domain, PBD: GTPase binding domain; Ser/Thr kinase: serine threonine kinase domain.
(TIF)

Figure S2 ***pix-1(ok982)*** **and** ***pak-1(ok448)*** **arrested larvae present severe pharynx pumping defects.** 48 hours after egg-laying, pharynx pumping rates were counted on arrested L1 animals and escaper L3 animals moving freely on a bacterial lawn. At least 10 animals per genotype were examined during 15-sec periods. N = 3. ** T-test *p* (mutant/N2)<0.001
(TIF)

Figure S3 **Establishment of embryo width measurement as a robust metrics to characterize embryonic elongation.** A) We tested the robustness and reproducibility of head width measurement of embryos. To do so, head width was measured five times on a given population of *wt* embryos at 1.2-fold stage (n = 12 embryos). Means and standard deviation were calculated and Brown-Forsythe test (using R statistical package) was used to test for homogeneity of variances among the five different groups of measurement. This test revealed no significant variance difference amongst the measurements (F-test p-value>0.5). B) The repeatability and batch effect of our measurements were assessed through measurement of the head width of *wt* embryos at 1.2-fold stage from 4D-recording done at three different days (n = 12 embryos). Means and standard deviation were calculated and Brown-Forsythe test was used to test for homogeneity of variances among the three different groups of measurements. This test revealed no significant variance difference amongst the measurements (F-test *p-value*>0.5). Similar results were obtained for tail width measurements and for measurement done at different stages of early elongation (data not shown). These data indicate that the significant differences observed between genotypes using head-width, tail-width and head/tail width ratio measurements are not due to measurement variability and batch effect.
(TIF)

Figure S4 **PIX-1 is homogeneously distributed in the cytoplasm and at the cell periphery of hypodermal cells during early elongation.** Confocal microscopy analysis of *pix-1(gk416)* embryos carrying *sajEx1[pix-1p::pix-1::gfp; rol-6]; mcIs40[lin-26p::ABDvab-10::mCherry + myo-2p::gfp]. PIX-1::GFP* is observed in B and E (green in C, F) and *VAB-10_ABD_::mCherry* in A and D (red in C, F). Embryos are oriented anterior to the left and dorsal up. Enlarged views (lower panels) show areas indicated by white rectangles in upper panels. Apical and basolateral membrane are indicated by arrow and arrowhead, respectively (L, lower panel). Scale bars upper panels: 10 µm; lower panels 5 µm.
(TIF)

Figure S5 **PIX-1::GFP intensity ratio are constant throughout early elongation.** Dorsal-posterior/dorsal-anterior (DP/DA), lateral-posterior/lateral-anterior (LP/LA), ventral-posterior/ventral-anterior (VP/VA), dorsal-posterior/ventral (DP/V), dorsal-anterior/ventral (DA/V) and dorsal-anterior/lateral (DA/L) fluorescence intensity ratio were measured as detailed in methods and in Figure 5 in *pix-1(gk416); unc-119; sajIs2[lin-26p::pix-1::GFP,unc-119^R^]* embryos during early elongation. Bar correspond to the mean and error bars to the standard deviation. * T-test<0.05
(TIF)

Figure S6 **Only DP/DA ratio inversely correlates with the elongation rate of the embryos during early elongation.** Scatter plot representing the relationship between the lateral-posterior/lateral-anterior (LP/LA), ventral-posterior/ventral-anterior (VP/VA), dorsal-posterior/dorsal-anterior (DP/DA) and dorsal-anterior/ventral (DA/V) intensity ratio of PIX-1::GFP and the elongation rate in μm/min during early elongation of *pix-1(gk416); sajEx1[pix-1p::pix-1::GFP, rol-6]* embryos (n = 20). The spearman correlation (R2) between the elongation rate and the PIX-1::GFP intensity ratio are indicated, as well as the *p-values* rejecting the null hypothesis being that the two values are not significantly correlated. Similar results were obtained from two independent transgenic lines.
(TIF)

Figure S7 **High expression of PIX-1::GFP in dorsal-posterior hypodermis is detrimental for elongation rate of embryos.** Dorsal-posterior/dorsal-anterior (DP/DA), lateral/ventral (L/V), dorsal-anterior/lateral (DA/L) and dorsal-posterior/lateral (DP/L) fluorescence intensity ratio were measured as detailed in methods and in Figure 5 in *pix-1(gk416); unc-119; sajIs2[lin-26p::pix-1::GFP;unc-119^R^]* embryos elongating at a *wt*-rate (elongation≥288 nm/min) or elongating slower (elongation<288 nm/min) during early elongation. Bar correspond to the mean and error bars to the standard deviation. ** T-test p-value<0.01.
(TIF)
