# Supplementary figures and images for: pix-1 Controls Early Elongation in Parallel with mel-11 and let-502 in Caenorhabditis elegans
Source: PLoS One. 2014 Apr 14;9(4):e94684. doi: 10.1371/journal.pone.0094684 (PMC3986101; doi:10.1371/journal.pone.0094684)

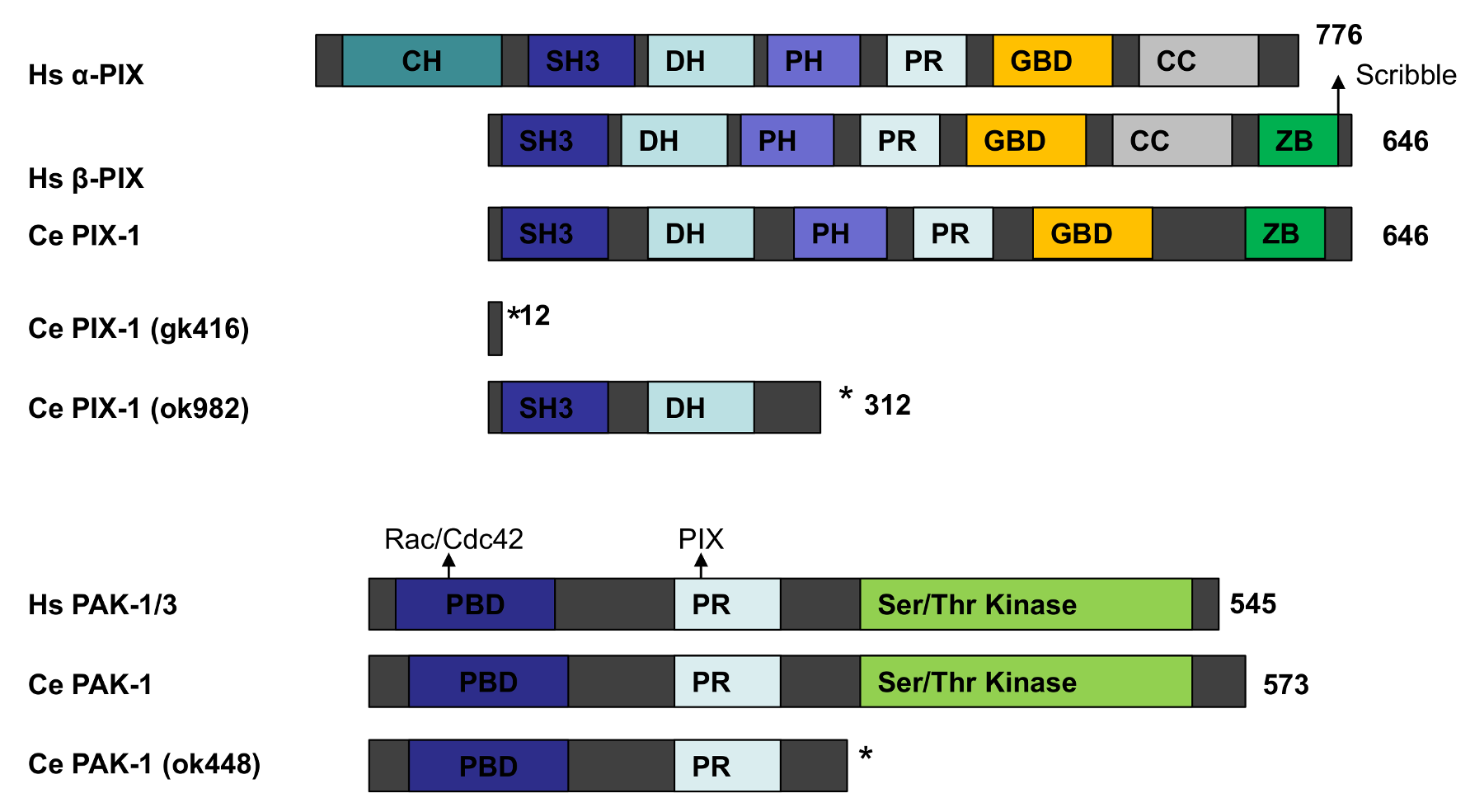

Supplement: Figure S1 — Schematic representation of human (Hs) and C. elegans (Ce) PIX and PAKs. Modular structure has been identified using the SMART tool (www.smart.org) or by alignment of consensus sequence using CLUSTALW. Binding-domains for protein partners reported in the literature are indicated. Proteins coded by C. elegans mutant alleles are indicated. * indicate the location of translation arrest. CH: calponin homology domain; SH3: src homology domain; DH: dbl homology domain; PH: Pleckstrin homology domain; PR: Proline Rich sequence, GBD: GIT-binding domain, CC: coil-coiled domain, ZB: PDZ binding domain, PBD: GTPase binding domain; Ser/Thr kinase: serine threonine kinase domain. (TIF) [file pone.0094684.s001.tif]

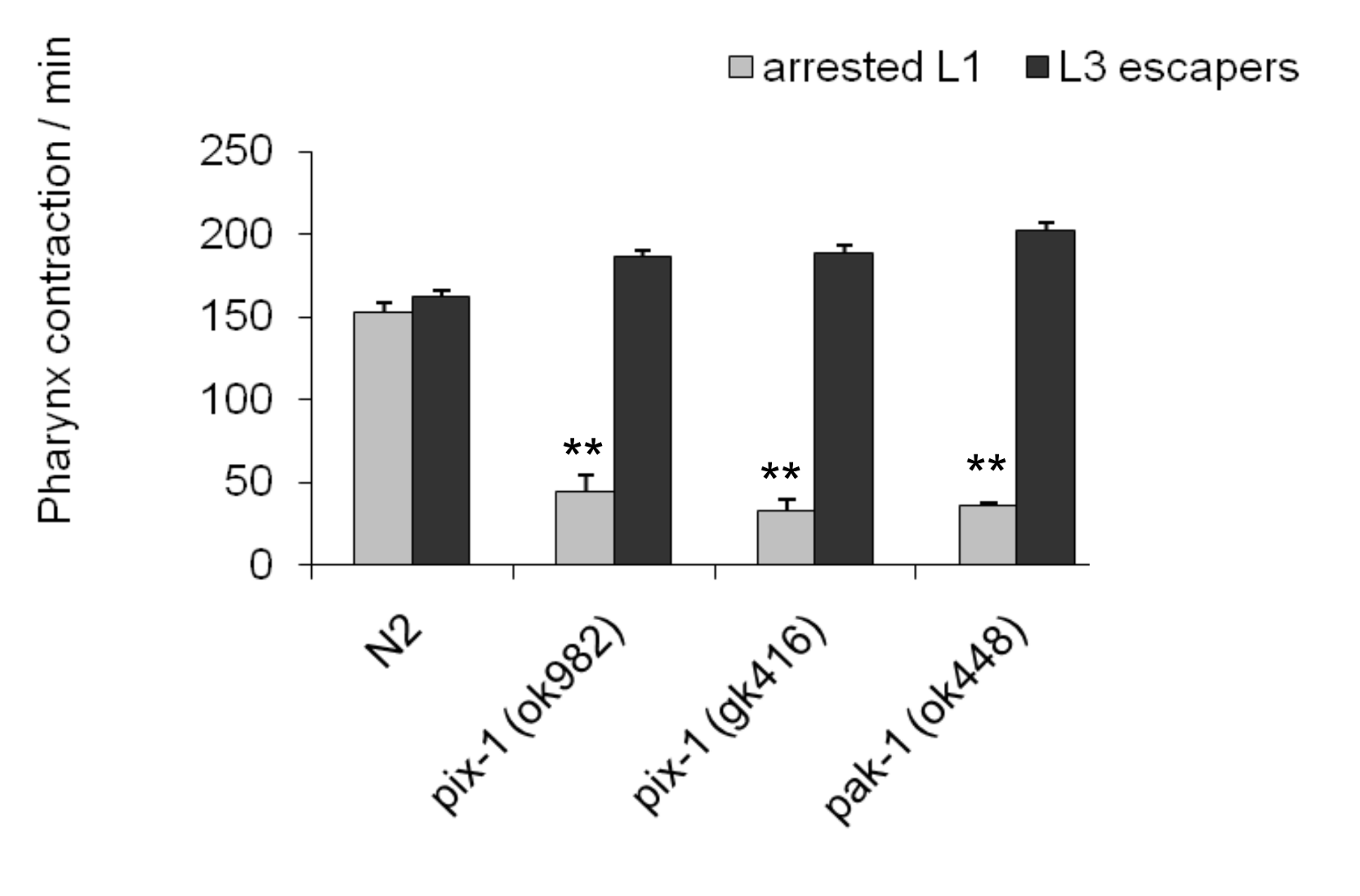

Supplement: Figure S2 — pix-1(ok982) and pak-1(ok448) arrested larvae present severe pharynx pumping defects. 48 hours after egg-laying, pharynx pumping rates were counted on arrested L1 animals and escaper L3 animals moving freely on a bacterial lawn. At least 10 animals per genotype were examined during 15-sec periods. N = 3. ** T-test p (mutant/N2)<0.001 (TIF) [file pone.0094684.s002.tif]

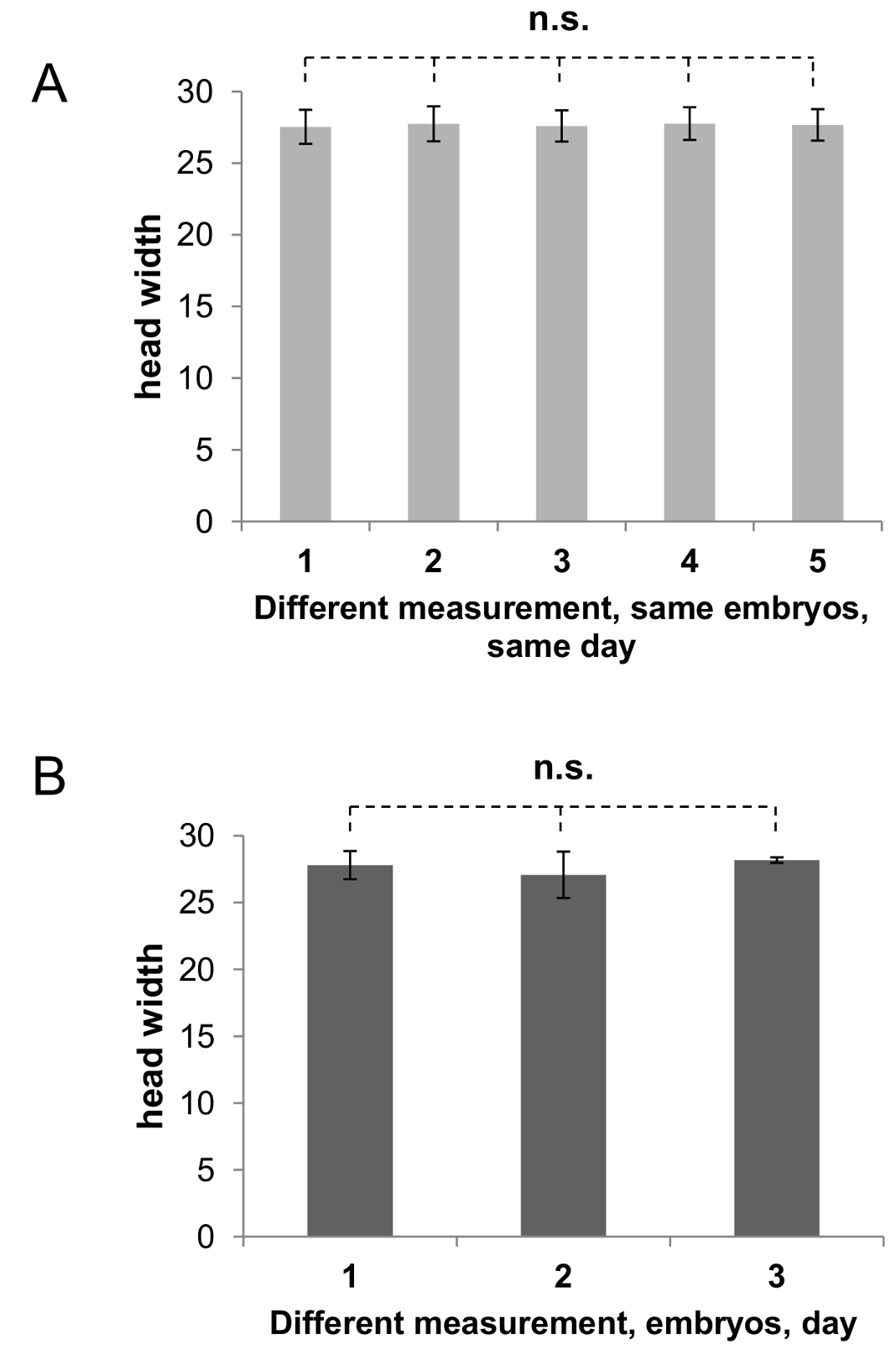

Supplement: Figure S3 — Establishment of embryo width measurement as a robust metrics to characterize embryonic elongation. A) We tested the robustness and reproducibility of head width measurement of embryos. To do so, head width was measured five times on a given population of wt embryos at 1.2-fold stage (n = 12 embryos). Means and standard deviation were calculated and Brown-Forsythe test (using R statistical package) was used to test for homogeneity of variances among the five different groups of measurement. This test revealed no significant variance difference amongst the measurements (F-test p-value>0.5). B) The repeatability and batch effect of our measurements were assessed through measurement of the head width of wt embryos at 1.2-fold stage from 4D-recording done at three different days (n = 12 embryos). Means and standard deviation were calculated and Brown-Forsythe test was used to test for homogeneity of variances among the three different groups of measurements. This test revealed no significant variance difference amongst the measurements (F-test p-value>0.5). Similar results were obtained for tail width measurements and for measurement done at different stages of early elongation (data not shown). These data indicate that the significant differences observed between genotypes using head-width, tail-width and head/tail width ratio measurements are not due to measurement variability and batch effect. (TIF) [file pone.0094684.s003.tif]

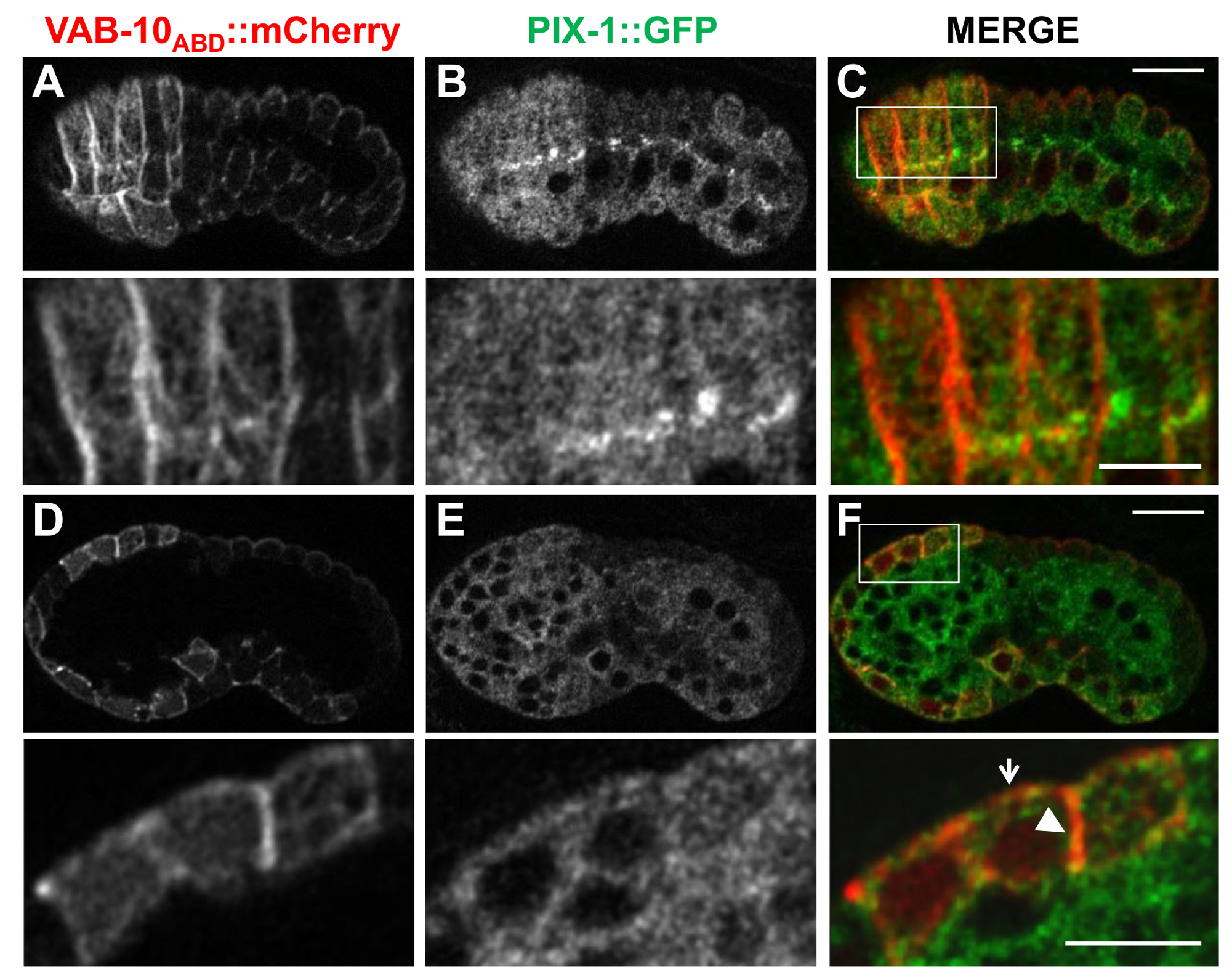

Supplement: Figure S4 — PIX-1 is homogeneously distributed in the cytoplasm and at the cell periphery of hypodermal cells during early elongation. Confocal microscopy analysis of pix-1(gk416) embryos carrying sajEx1[pix-1p::pix-1::gfp; rol-6]; mcIs40[lin-26p::ABDvab-10::mCherry + myo-2p::gfp]. PIX-1::GFP is observed in B and E (green in C, F) and VAB-10ABD::mCherry in A and D (red in C, F). Embryos are oriented anterior to the left and dorsal up. Enlarged views (lower panels) show areas indicated by white rectangles in upper panels. Apical and basolateral membrane are indicated by arrow and arrowhead, respectively (L, lower panel). Scale bars upper panels: 10 µm; lower panels 5 µm. (TIF) [file pone.0094684.s004.tif]

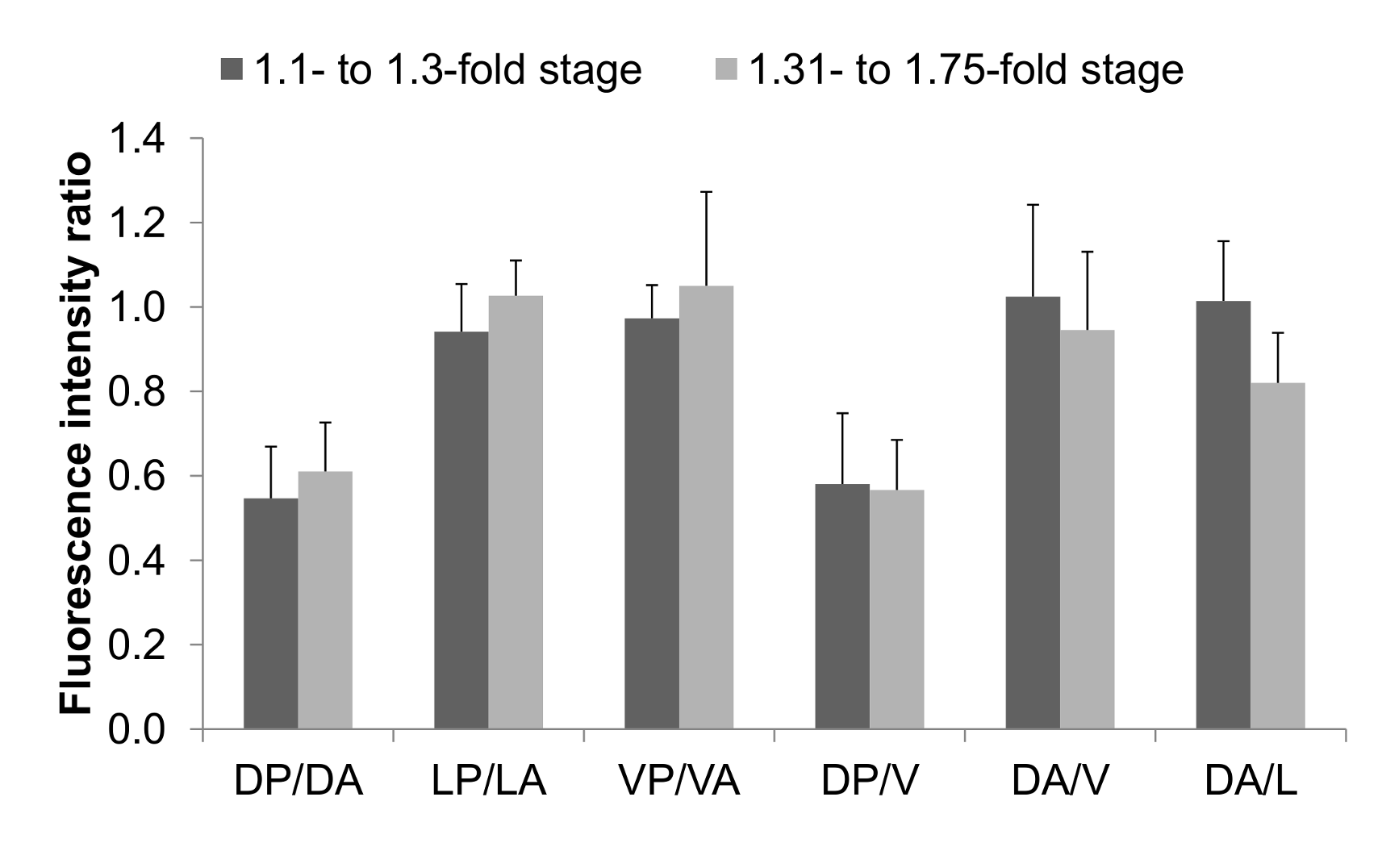

Supplement: Figure S5 — PIX-1::GFP intensity ratio are constant throughout early elongation. Dorsal-posterior/dorsal-anterior (DP/DA), lateral-posterior/lateral-anterior (LP/LA), ventral-posterior/ventral-anterior (VP/VA), dorsal-posterior/ventral (DP/V), dorsal-anterior/ventral (DA/V) and dorsal-anterior/lateral (DA/L) fluorescence intensity ratio were measured as detailed in methods and in Figure 5 in pix-1(gk416); unc-119; sajIs2[lin-26p::pix-1::GFP,unc-119R] embryos during early elongation. Bar correspond to the mean and error bars to the standard deviation. * T-test<0.05 (TIF) [file pone.0094684.s005.tif]

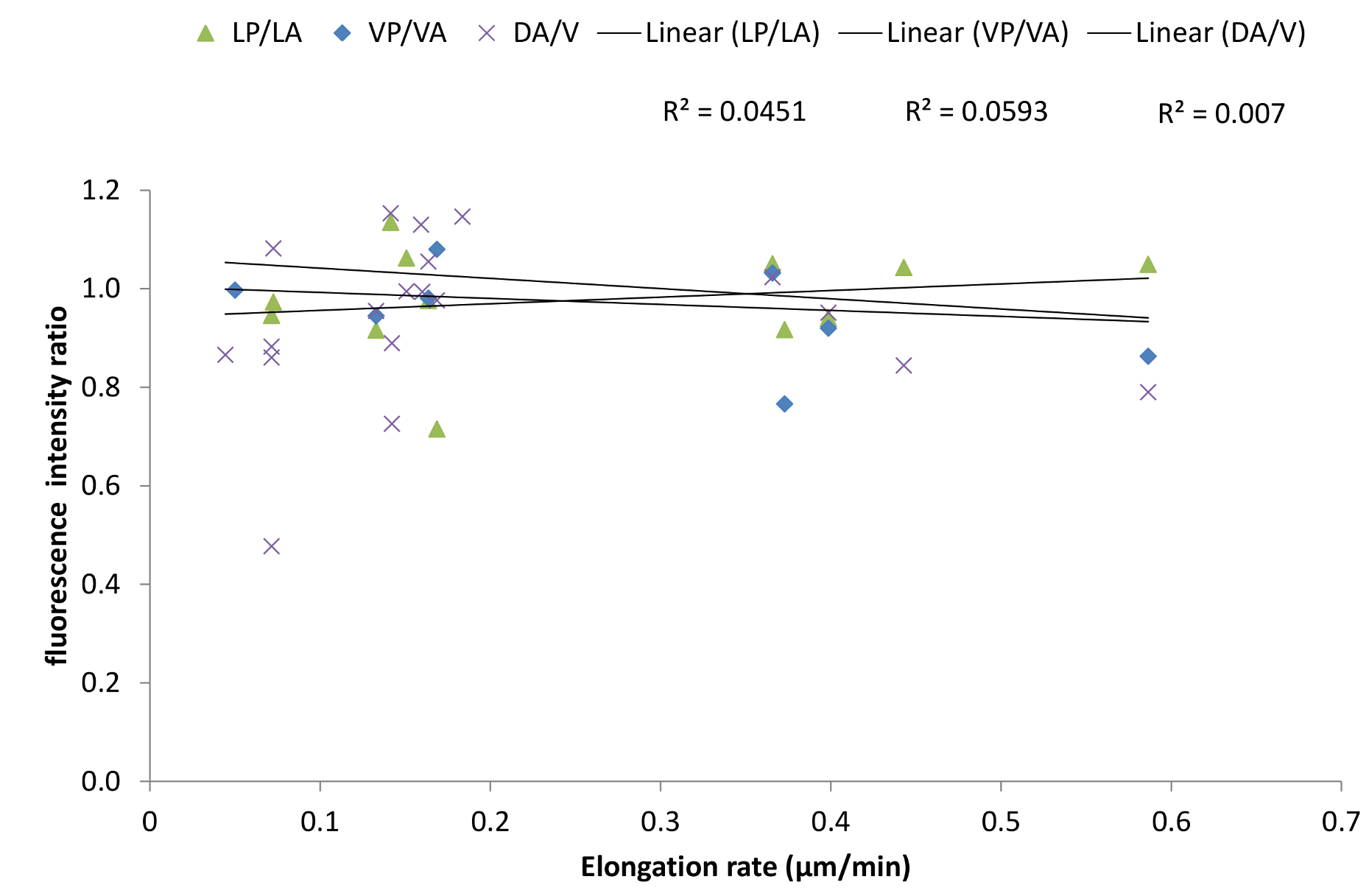

Supplement: Figure S6 — Only DP/DA ratio inversely correlates with the elongation rate of the embryos during early elongation. Scatter plot representing the relationship between the lateral-posterior/lateral-anterior (LP/LA), ventral-posterior/ventral-anterior (VP/VA), dorsal-posterior/dorsal-anterior (DP/DA) and dorsal-anterior/ventral (DA/V) intensity ratio of PIX-1::GFP and the elongation rate in μm/min during early elongation of pix-1(gk416); sajEx1[pix-1p::pix-1::GFP, rol-6] embryos (n = 20). The spearman correlation (R2) between the elongation rate and the PIX-1::GFP intensity ratio are indicated, as well as the p-values rejecting the null hypothesis being that the two values are not significantly correlated. Similar results were obtained from two independent transgenic lines. (TIF) [file pone.0094684.s006.tif]

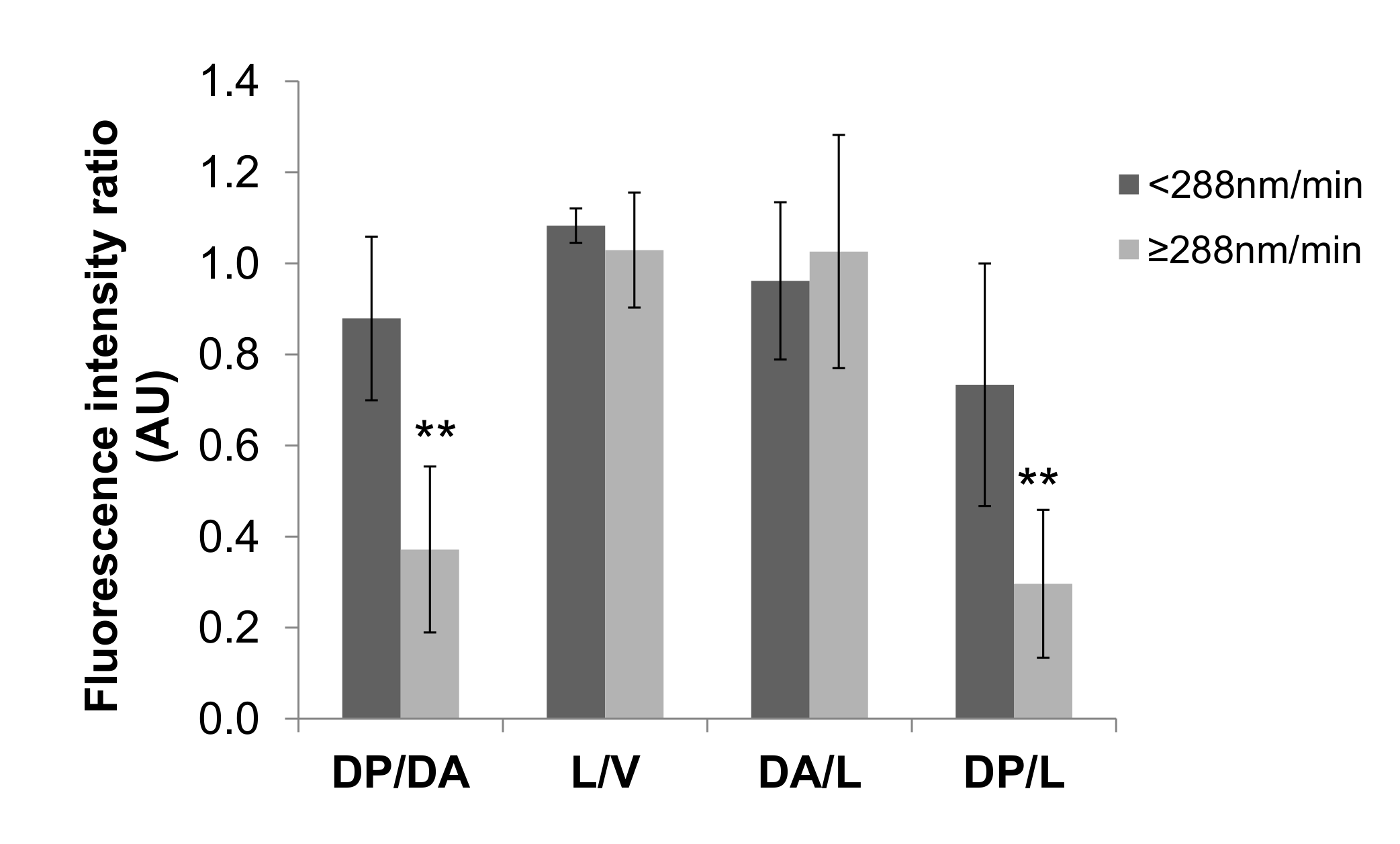

Supplement: Figure S7 — High expression of PIX-1::GFP in dorsal-posterior hypodermis is detrimental for elongation rate of embryos. Dorsal-posterior/dorsal-anterior (DP/DA), lateral/ventral (L/V), dorsal-anterior/lateral (DA/L) and dorsal-posterior/lateral (DP/L) fluorescence intensity ratio were measured as detailed in methods and in Figure 5 in pix-1(gk416); unc-119; sajIs2[lin-26p::pix-1::GFP;unc-119R] embryos elongating at a wt-rate (elongation≥288 nm/min) or elongating slower (elongation<288 nm/min) during early elongation. Bar correspond to the mean and error bars to the standard deviation. ** T-test p-value<0.01. (TIF) [file pone.0094684.s007.tif]
